# Root Growth Adaptation to Climate Change in Crops

**DOI:** 10.3389/fpls.2020.00544

**Published:** 2020-05-08

**Authors:** J. Calleja-Cabrera, M. Boter, L. Oñate-Sánchez, M. Pernas

**Affiliations:** Centro de Biotecnología y Genómica de Plantas, Universidad Politécnica de Madrid – Instituto Nacional de Investigación y Tecnología Agraria y Alimentaria, Madrid, Spain

**Keywords:** climate change, root traits, crop yield, adaptation, increased temperature

## Abstract

Climate change is threatening crop productivity worldwide and new solutions to adapt crops to these environmental changes are urgently needed. Elevated temperatures driven by climate change affect developmental and physiological plant processes that, ultimately, impact on crop yield and quality. Plant roots are responsible for water and nutrients uptake, but changes in soil temperatures alters this process limiting crop growth. With the predicted variable climatic forecast, the development of an efficient root system better adapted to changing soil and environmental conditions is crucial for enhancing crop productivity. Root traits associated with improved adaptation to rising temperatures are increasingly being analyzed to obtain more suitable crop varieties. In this review, we will summarize the current knowledge about the effect of increasing temperatures on root growth and their impact on crop yield. First, we will describe the main alterations in root architecture that different crops undergo in response to warmer soils. Then, we will outline the main coordinated physiological and metabolic changes taking place in roots and aerial parts that modulate the global response of the plant to increased temperatures. We will discuss on some of the main regulatory mechanisms controlling root adaptation to warmer soils, including the activation of heat and oxidative pathways to prevent damage of root cells and disruption of root growth; the interplay between hormonal regulatory pathways and the global changes on gene expression and protein homeostasis. We will also consider that in the field, increasing temperatures are usually associated with other abiotic and biotic stresses such as drought, salinity, nutrient deficiencies, and pathogen infections. We will present recent advances on how the root system is able to integrate and respond to complex and different stimuli in order to adapt to an increasingly changing environment. Finally, we will discuss the new prospects and challenges in this field as well as the more promising pathways for future research.

## Climate Change and Crop Yield

Effects of climate change are accelerating significantly since the last century. Changes in weather conditions and increases in the occurrence of extreme events are being felt more often. The Earth’s climate continues to warm and, all the model simulations predict a global trend to warmer temperatures (Lean and Rind, 2009). Considering the temperature data, the northern hemisphere is warming more rapidly than the southern hemisphere (Foster and Rahmstorf, 2011). Although long term weather changes are more difficult to predict, it is expected that, by 2050, the global mean temperature increase 1.5–2°C. These changes in the global temperature would cause further alterations in the climate leading to an increased frequency of heat-waves, fewer days of freezing temperatures, less rainfall but more intense precipitations and higher incidence of droughts and other weather extremes experienced across the globe that will negatively affect agricultural production (Easterling et al., 2000; Dempewolf et al., 2014). The global population is expected to reach nine billion by 2050, representing an additional two billion people to feed (Ray et al., 2013). The projections show that feeding world’s population would require raising the overall food production by around 70% by 2050 (Global agriculture toward 2050. Rome, FAO,2009a). However, current trajectory shows that the rates of global production in key crops would increase far below what is needed to produce enough food to meet the raising population demands (Ray et al., 2013). This widening mismatch between demand and supply is causing concern for future food security (Godfray et al., 2010). Further reasons for alarm are the yield losses predicted to be provoked by climate change (Lobell et al., 2011; Tai et al., 2014). Although climate changes will not impact crop production evenly according to geographical distribution, it will threaten food production globally (Thiault et al., 2019). For all those reasons, there is an urgent need to maintain and improve crop productivity under these climatic constrains (Bailey-Serres et al., 2019; Shan-e-Ali Zaidi et al., 2019).

### Climate Change Impact on Crops

Climate change is a long-term challenge, but requires urgent action given the pace and the scale by which greenhouse gases are accumulating in the atmosphere and the risk of more than 2°C global temperature rise. Greenhouse gases (CO_2_, O_3_, and CH_4_) driving climate change, affect directly crop productivity (IPCC, 2014). Higher concentrations of CO_2_ are expected to act as a fertilizer by improving net photosynthesis rates and increasing water use efficiency (Long et al., 2004; Long et al., 2004; Deryng et al., 2016). This positive effect is higher in C3 plants such as wheat, rice and soybean, due to the limited photosynthetic output of photorespiratory carbon losses. Nevertheless, in the long term, the constant increment of CO_2_ concentration will have a negative impact in the climate, thus counterbalancing the increase in crop yield (Specht et al., 1999; Long et al., 2004; Dong et al., 2018; Senapati et al., 2019; Wei et al., 2019). On the other hand, O_3_ changes have significant negative effects on the yield of major agricultural crops. O_3_ is one of the most highly reactive oxidants, provoking damage in plant tissues, which includes visible leaf injuries, decreased photosynthesis and accelerated senescence and cell death (Vandermeiren et al., 2009). But interestingly, there are pronounced differences in O_3_ sensitivity between species (Mills et al., 2007). O_3_ causes a decrease in crop biomass in wheat and soybean, more specifically root biomass, during reproductive and grain filling stages leading to a reduction of overall crop yield. Consequently, global production losses due to O_3_ in these crops are expected to be higher than losses in rice and maize (Van Dingenen et al., 2009; Avnery et al., 2011; Tang et al., 2013; Feng Z. et al., 2019; Wang Y. et al., 2019).

Climate change is causing the shifting of the rainfall patterns. More intense rainfall producing flooding periods, the appearance of drought seasons and offseason precipitations are expected. In several prediction models, offseason rainfall during critical stages of crop growing could lead to a very significant reduction in crop yield (Lobell and Burke, 2008). In winter oilseed rape it has been reported that a more intense rainfall during autumn and winter periods may boost the appearance of diseases (Sharif et al., 2017). And in maize and soybean, more intense precipitations in spring provoke early damage in young plants (Urban et al., 2015). Another risk associated to more extreme rainfall is the intensification of flooding events. In China or Bangladesh much of the harvest areas are in the flooding threatened regions. Floods put in danger the food security of these countries by destroying cropping areas or delaying crop planting due to high soil moisture (Monirul Qader Mirza, 2002; Xu et al., 2013; Iizumi and Ramankutty, 2015). Moreover, in coming years the flooding risk of coastal regions will increase due to the rising of the sea-level and the alteration of climatology. Seawater flooding of coastal regions is becoming more frequent because waves and storm surges are getting stronger (Vitousek et al., 2017). Osmotic and anionic stress caused by the high salinity of seawater will become an additional problem to crops besides the low O_2_ and CO_2_ levels caused by anoxia. It has been shown that oilseed rape plants exposed to seawater flooding conditions suffer a reduction in plant biomass and a fall in productivity due to a lower number of siliques per plant and a lower seed mass (Hanley et al., 2019).

More frequent drought events are also expected due to longer periods without rain added to warmer temperatures. Although droughts restrict cropping areas, the decrease of agricultural productivity is mainly caused by a severe direct effect on crop yield (Saadi et al., 2015; Lesk et al., 2016; Zipper et al., 2016). The most damaging impact of drought stress on crop productivity occurs at reproductive or growing stages. The former produces pollen sterility (as observed in barley) or ovary abortion (as observed in maize) and the latter a reduction in kernel number and biomass (Boyer and Westgate, 2004). In general, a drought period causes a reduction of water consumption by the plant, leading to a stomatal closure and lower CO_2_ intake. Following decrease in photosynthesis ratio provokes a final reduction of crop biomass (Garofalo et al., 2019). The water scarcity imposed by drought is frequently accompanied by salinity stress. The ion toxicity and the reduction of soil water potential contribute to a severe reduction of plant growth. Soil salinity reduces yield in highly tolerant crops as cotton, barley and sugar beet as well as in crops with high salinity sensitivity as sweet potato, wheat or maize (Zörb et al., 2019).

**FIGURE 1:**
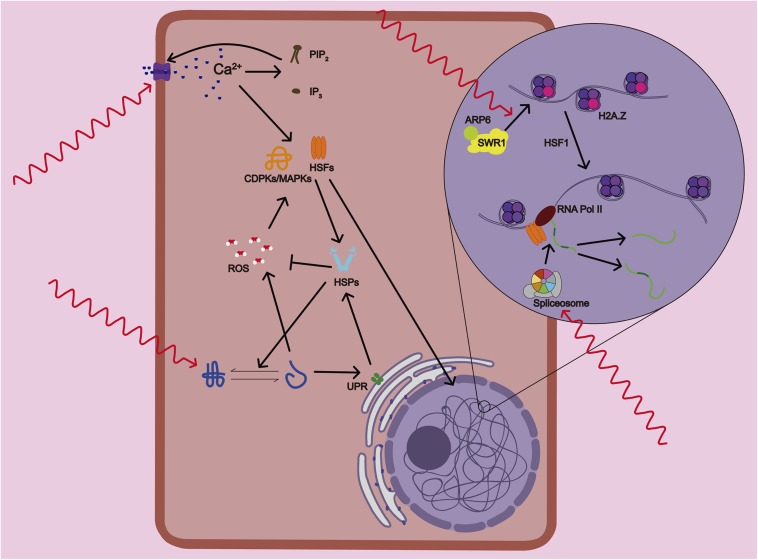
Mechanisms of temperature sensing and response in plants. Plants sense variations in temperature that are translated into the activation of several physiological and signaling processes. Primary temperature-sensing events start with the alteration of membrane fluidity and composition that causes the activation of calcium (Ca^2+^) channels. A feedback mechanism between the calcium and lipid signaling through accumulation of PIP_2_ and IP_3,_ enhances even further the Ca^2+^ entry in the cell. Several heat shock transcription factors (HSFs) and calcium-dependent protein kinases (CDPKs and MAPKs) are activated by Ca^2+^ and ROS/redox signaling network. At the same time, the accumulation of unfolded proteins in the endoplasmic reticulum (ER) that are potentially toxic activates the ER stress that sets off the unfolded protein response (UPR), a cytoprotective signaling pathway. Subsequent activation of bZIP transcription factors induces the expression of Heat Shock Proteins (HSPs). HSPs protect proteins from misfolding and subsequent loss of functionality and help the detoxification of ROS. ARP6, a subunit of SWR1 complex, mediates the insertion of the variant histone H2A.Z in the nucleosome. At warmer temperatures, the antagonistic roles of H2A.Z and HSF1 seem to be required to activate heat response (HR) gene transcription. Lastly, the alternative splicing machinery allows the rapid adjustment of the abundance and function of key stress-response components.

All these adverse climate effects together with elevated temperature will increase agriculture losses even further (Fuhrer, 2003; Lobell and Burke, 2008; Ainsworth, 2017; Tai and Val Martin, 2017). Numerous studies suggested that global warming will lead to substantial declines in mean crop yields in the next future, and that the most serious agricultural impacts will occur in the tropics, where the majority of the world’s food-insecure population resides (Battisti and Naylor, 2009). Furthermore, mean crop yield will decline and their variability will increase even if interannual climate variability remains unchanged (Tigchelaar et al., 2018). Adding up these and other effects, models show possible yield losses of 6–10% per 1°C of warming in the average temperature of the growing season (Guarino and Lobell, 2011). Moreover, climate variation is already causing a major effect on the stability of crop production. Yields of the top ten global crops–barley, cassava, maize, oil palm, rapeseed, rice, sorghum, soybean, sugarcane and wheat has been affected significantly in different regions all over the world (Ray et al., 2019). In this review we will focus on the effect and consequences of one of the major components of climate change, increased temperature and, in particular, its effect on crop roots (Figure 1).

### Increased Temperature Impact on Crops

As a consequence of global warming, the yield increment that started in the last century is stagnant and even decreasing in some areas (Lobell and Field, 2007). High temperature response has been studied at extreme conditions characterized by the heat shock response. However, even small differences in ambient growth temperature can have profound effects on crop growth and yield. Although abundant literature is available on how plants tolerate extreme damaging heat less is known on how crops adapt to moderately increased or warmer temperatures (Quint et al., 2016; Vu et al., 2019b).

Prediction models reveal that the continuous increment in temperature would result in heavy losses in crop yield at medium latitudes (Liu et al., 2016), whereas less fertile soil areas located at extreme latitudes are getting a more appropriate climate for agriculture (Long and Ort, 2010; Lobell et al., 2011; Iizumi and Ramankutty, 2015; Sharif et al., 2017). Thus, warmer temperature could expand the areas potentially suitable for cropping, increase the length of the growing period, and crop yields may rise in these areas (How to Feed the World in 2050, Rome; FAO, 2009b). However, globally higher temperatures shorten the growth season, letting the crops with a much shorter period to perform photosynthesis even in the case of well irrigated and tolerant crops. Moreover, heat stress directly affects photosynthetic rate accentuating the effect of this shorter growth period. As a result, crops have less biomass to face the anthesis and the consequent grain filling. Warmer environments also affect post-anthesis stages reducing grain growth and promoting fruit senescence. Additionally, the increase in temperature promotes a higher evapotranspiration rate that, ultimately reduce soil moisture and the available water needed for grain filling. When plants suffer extreme temperatures of short duration these processes are even more severely affected (Asseng et al., 2011, 2015, 2019; Liu et al., 2014, 2019; Lesk et al., 2016). Accordingly, it has been reported that in wheat, rice and shorghum heat causes loss of grain yield by shortening its growth period, altering spikelet’s development (number of spikes per plant and spikes size), grains per spike and reducing grain size (Prasad et al., 2006; Jagadish et al., 2010; Fahad et al., 2017). Similarly, in oilseed rape, *Brassica rapa* and *Brassica juncea* yield losses are produced by a decrease in seeds per silique and number of siliques per plant as well as defects in pod formation (Angadi et al., 2000; Morrison and Stewart, 2002). High temperatures also lead to a decrease in crop quality by changing seed composition. Thus, in cereals and oilseed crops heat stress reduces the oil, starch, and protein contents of seeds (Jagadish et al., 2015; Fahad et al., 2017). It has been shown that in wheat, increased temperatures reduce the levels of valuable protein whereas it causes the accumulation of proline and soluble carbohydrates (Qaseem et al., 2019). On the other hand, higher temperature also reduces oilseed rape seeds quality by reducing the amounts of oil and increasing the levels of proteins and glucosinolates (Aksouh et al., 2001). In rice, high temperatures during ripening led to the deterioration of grain quality including starch accumulation (Morita et al., 2016; Chen et al., 2017). In brief, crops are substantially but heterogeneously affected by temperature variability (Thiault et al., 2019). To remedy this effect, we need to evaluate and understand further the changes that crops undergo under the future climatic scenario.

## Root Response to Increased Temperature

Crops face rising temperatures by triggering a heat response, whose timing and effectiveness will determine if the plants overcome the stress. The effect of increased temperatures on aerial parts of the plants and their responses has been well studied, whereas their influence and response on roots (and root-to-shoot signaling) has been less explored (Wahid et al., 2007). If we attempt to enhance adaptation of crops to severer environments triggered by climate change, we need to take into account below ground traits. For that, first, we need to improve our understanding of the processes regulating the root response to increased temperature.

Plants have a greater water demand in warmer environments due to increased water loss by evapotranspiration and decreased water uptake by the root, causing an overall water deficit situation (Heckathorn et al., 2013). Water uptake takes place in the root either through aquaporins, membrane channels that facilitates water transport inside the cells, or by diffusion through plasmatic membrane (Maurel et al., 2015). Studies with several crops have shown different response of aquaporins and plasmatic membrane fluidity to higher temperatures in roots. Thus, in pepper and wheat, water uptake in warmer soil seems to positively correlate with aquaporin activity (Carvajal et al., 1996; Cabañero et al., 2004), whereas in broccoli (*Brassica oleracea* var. *italica*) and maize, warmer temperatures decrease aquaporin quantity and activity but increase membrane fluidity. When temperature is extreme, the membrane starts to rigidify heavily decreasing even more water uptake (Iglesias-Acosta et al., 2010; Ionenko et al., 2010).

Nutrient balance is also altered by changes in temperature. Similarly to water, temperature effect on nutrient uptake varies depending on the crop. In tomato, warmer soils limit root growth and decrease nutrient uptake causing a reduction in macro and micro-nutrient levels (Tindall et al., 1990; Giri et al., 2017). In *Agrostis stolonifera*, a grass species used as fodder for livestock, the application of high temperature to roots results in a lower number of roots and an increase in the uptake and partitioning of nitrogen, phosphorous and potassium (Huang and Xu, 2000). In *Andropogon gerardii*, another plant used as fodder, supra-optimal root temperatures cause a decrease in root and shoot growth. Further higher temperatures moderately affects nitrogen uptake but its efficiency use is severely perturbed (DeLucia et al., 1992). In contrast, warm temperature does not alter nitrogen, phosphorus and potassium uptake in maize, but higher temperatures seem to only slightly decrease phosphorus and potassium uptake (Bravo-F and Uribe, 1981; Hussain et al., 2019).

All these negative root responses to increase temperature severely compromise water and nutrient uptake and the consequence is a dramatic reduction on crop yield. Cultivars better adapted to temperature will have to shape their roots to improve their water and nutrient efficiency if they aim to secure yield stability under this challenging environment. As we will ascertain during this review, root organization shows a high plasticity in response to soil changes providing high opportunities for improvement. Better comprehension of the physiological, genetic and molecular mechanisms regulating this plasticity will allow us to develop better adapted crops.

### Temperature Sensing and Signaling in Roots

**FIGURE 2:**
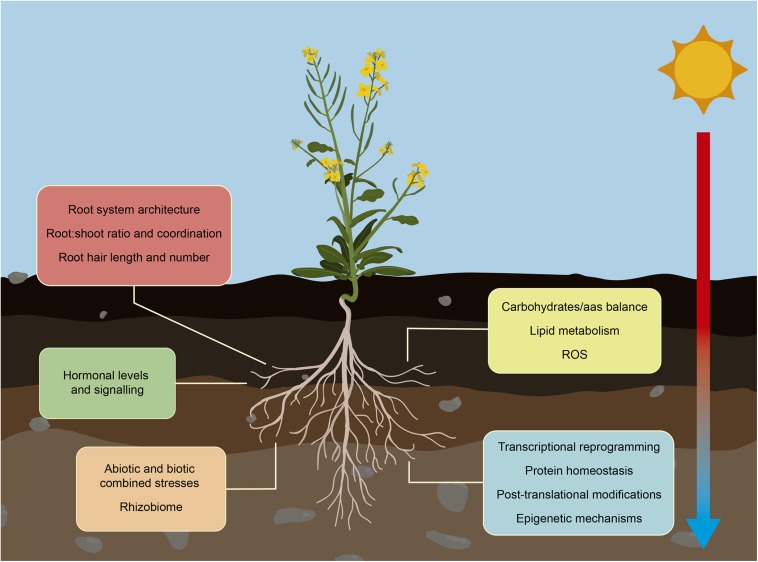
Root response to increased ambient temperature. Climate change is increasing the ambient temperature altering crops growth. Crops adapt root development and functionality to maintain water and nutrients availability in this stressing environmental situation. These changes in their RSA, include alterations in lateral and primary root growth and root hair elongation, and adjustment of their interchange with aboveground organs. Roots also suffer changes in their metabolism affecting mainly carbohydrate/amino acid balance, lipid metabolism and the activation of heat and oxidative pathways to prevent disruption of root growth. Temperature-mediated alteration of hormone levels trigger signal transduction pathways that prepare plants to overcome the stress situation. Other significant molecular changes that regulate root adaptation include global transcriptomic reprogramming, changes in protein profiles, and activation of epigenetic and chromatin-based mechanisms. In the field, increasing temperature is usually accompanied with other abiotic and biotic stresses such as drought, salinity, nutrient deficiency and pathogen infections. Roots are able to integrate and respond to all these different stress situations to promote their survival and maintain their growth.

Although it has been proposed that thermomorphogenesis signaling could differs between roots and shoots, a common set of mechanisms of temperature sensing mediate organ response at a molecular and cellular level (Bellstaedt et al., 2019). Plants can sense small variations in temperature, and this sensing can be translated into activation of several physiological processes that are considered the primary temperature-sensing events (Figure 2; Penfield, 2008; McClung and Davis, 2010). Roots sense these thermal changes directly or indirectly. Indirectly sensing is either triggered by the shoot demand of water and nutrient or by the supply of carbon from the shoot to root (Plieth et al., 1999; Heckathorn et al., 2013). Warmer temperatures, and more sharply, high temperature, alter the stability of membranes and cystoskeleton components, as well as proteins and nucleic acids (Vu et al., 2019a). Temperature changes alter membrane fluidity and composition causing the activation of calcium (Ca^2+^) channels. Increased intracellular Ca^2+^ triggers the lipid signaling through the lipid-modifying enzymes PLD and PIPK. Subsequent accumulation of PIP_2_ and IP_3,_ in turn, enhances Ca^2+^ entry in the cell (Mittler et al., 2012). The Ca^2+^ influx can activate several heat shock transcription factors (HSFs) and calcium-dependent protein kinases (CDPKs and MAPKs) that control heat stress responses. The ROS/redox signaling network is also mediating plant sensing to high temperature due to direct activation of HSFs and heat related MAPKs. ROS accumulation might be produced as unwanted products of several metabolic pathways due to heat-mediated changes in the stability and activity of their enzymes or by calcium activation of ROS-producing enzyme RBOHD (Suzuki et al., 2011; Rasul et al., 2017).

Heat stress causes accumulation of unfolded proteins in the endoplasmic reticulum (ER) that are potentially toxic leading to what is known as ER stress. ER stress elicits the unfolded protein response (UPR), a cytoprotective response to mitigate and to protect from this damage (Howell, 2013). The UPR is signaled through two pathways: one involving the proteolytic processing transcription factor bZIP28, and the other involving the ribonuclease IRE1, which mediates the splicing of the bZIP60 transcription factor mRNA (Neill et al., 2019). Both UPR pathways induce the expression of Heat Shock Proteins (HSPs) and activation of brassinosteroids (BRs) signaling (Che et al., 2010). These two pathways seems to be less sensitive than Ca^2+^ channels because only high temperatures are able to provoke a global unfolding of proteins (Liu and Howell, 2016). HSPs are actively translated during the onset of temperature stress response to protect proteins from misfolding and subsequent loss of functionality. But HSPs also improves membrane stability and detoxification of ROS by regulating several antioxidant enzymes therefore attenuating stress response (Ul Haq et al., 2019).

ARP6, a subunit of SWR1 complex, has been proposed as a histone themosensor. ARP6 mediates the insertion of the variant histone H2A.Z in the nucleosome. H2A.Z nucleosomes wrap DNA more tightly, which affects the ability of RNA polymerase (Pol) II to initiate transcription. At warmer temperatures, H2A.Z is evicted from the nucleosomes located at the transcriptional start of heat response genes (Kumar and Wigge, 2010). This process also required the recruitment of HSFA1 to the promoters of these genes to activate their transcription (Cortijo et al., 2017). Therefore, the antagonistic roles of H2A.Z and HSF1 seems to be require to activate gene expression rapidly and precisely in response to elevated temperature (Wigge, 2013). Lastly, warmer temperature could alter RNA unfolding, metabolism and structure (Su et al., 2018) as well as changes in small RNA expression (Liu et al., 2017). It also causes a recruit of alternative splicing (AS) machinery that will allow the rapid adjustment of the abundance and function of key stress-response components (Laloum et al., 2018). All these pathways trigger different sensing events that contribute to the activation of the overall heat response. This heat response includes a large number of morphological, physiological, metabolic and molecular changes altering root growth that we will describe in more detail.

### Morphological and Physiological Response

**FIGURE 3:**
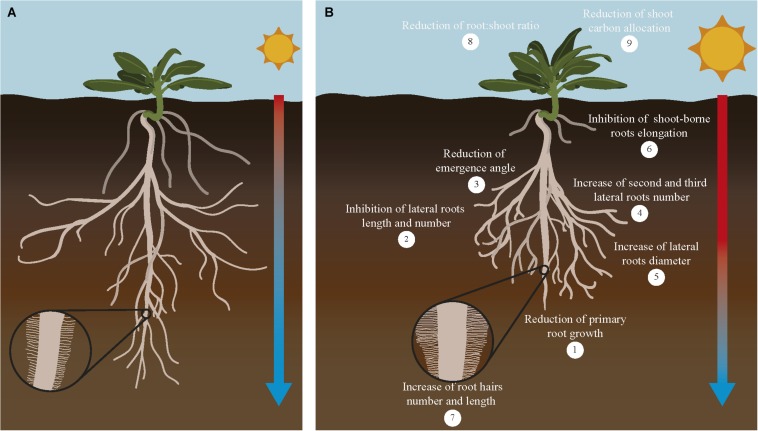
Response of major root traits to increasing temperatures in crops. Increasing temperature of the soil affects root traits related with its organization, growth and function. Root system architecture defined as the organization of the primary, lateral, shoot-borne and lateral roots is drastically altered in response to increased temperature in the soil **(B)** compared to plants growing in optimal conditions **(A)**. Crops growing under higher temperatures show shorter primary roots (1), reduction of lateral roots growth and number (2) and their angle of emergence (3), higher number of second and third order roots (4) with larger diameter (5), inhibition of shoot-borne roots (adventitious and nodal roots) elongation and number (6) and increase of root hairs number and length (7). In addition, this overall reduction on root system growth causes a reduction of root:shoot ratio (8) and reduction of root carbon allocation (9). As a consequence of all these changes, nutrient and water uptake conducted by the roots for the whole plant is compromised and crop yield is severely affected. Although most of these effects are detrimental to root growth, some responses alleviate this situation by increasing root:soil surface [increase in number of second to third roots number (4) and number and length of root hairs (7)], improving water efficiency uptake [increase in diameter of roots (5)], or increase in root depth (lower root angle). Interestingly, these root responses coincide with root traits associated with cultivars more tolerant to high temperatures. A comprehensive evaluation of these traits and their impact on crops productivity will help to decide which root traits are more valuable to be incorporated to breeding programs designed to improved crop yield under climate change conditions.

Roots need an optimal temperature range to have a proper growth rate and function. In general, optimal root temperature tends to be lower than optimal shoot temperature. Crop roots have different optimal root temperature depending on the species. Within this range, a higher temperature is usually associated to altered root:shoot ratio, and a further increase in temperature would limit root development and alter root system architecture (RSA) reducing root:shoot ratio (Ribeiro et al., 2014; Koevoets et al., 2016). RSA is defined as the organization of the primary, lateral, adventitious and accessory roots. Each RSA is determined by parameters such as length, number and angle of these root components. RSA is the main factor that controls nutrient and water uptake efficiency since it determines the soil volume that roots are able to explore at different environmental situations (Lynch, 1995). Generally, the exposure of roots to temperatures higher than the optimal causes a decrease in the primary root length, number of lateral roots and their angle of emergence. Moreover, the increase in temperature causes the initiation of second and third order lateral roots that are characterized by a larger diameter (Figure 3). The negative effect of increasing temperatures usually reduces the surface between root and soil, therefore decreasing nutrient and water uptake (Nagel et al., 2009). In cassava and sweet potato, high root zone temperature significantly decreases the total length of the adventitious roots and the number and total length of the first order lateral roots (Pardales et al., 1999). Seminal and crown roots retarded their emergence and elongation when wheat seedlings are grown at high temperature (Huang et al., 1991a). In maize adult plants, the increase in temperature slows down lateral root growth to promote the development of long axile roots to reach the water of the deeper soil layers (Hund et al., 2008). But in potato, the increase in temperature causes the inhibition of adventitious and lateral roots initiation and elongation. Other effects of the warmer soil in potato are the swelling of the root cap meristem and bending of the root tip. Alteration of root growth in these crops seems to be caused by a decrease in the cell division rate (Sattelmacher et al., 1990; Joshi et al., 2016). Similarly, in sorghum, high root zone temperature reduces the elongation and cell production rate in seminal roots (Pardales et al., 1992). Interestingly, in wheat the increase in temperature causes a decrease in the length and number of central late metaxylem in the root tip. This change has been interpreted as an adaptation to limit damage in the root by the changes in water viscosity and root hydraulic conductance produced by heat (Huang et al., 1991b; Morales et al., 2003).

Another strategy used by roots to cope with changing environmental conditions that affect water and nutrient availability is increasing the number of root hairs and their length. This increase enhances root surface area that in turn will improve soil exploration, and therefore, water and nutrient uptake (Pregitzer et al., 2000). Hence, the contribution of root hairs to total root surface area in two crops, oilseed rape and barley increases with temperature. This increase provides their roots with a greater surface area for absorption per unit root weight or length (Macduff et al., 1986). In Arabidopsis and soybean, the lack of root hairs produces reduction in heat adaptation competence suggesting a key role of root hairs in short-term adaptation to high temperatures (Tanaka et al., 2014; Valdés-López et al., 2016). Moreover, since genes that participate in early sensing and adaptation to high temperature are switched off in barley root-hairless mutant plants, it has been suggested a role of root hairs as sensors of environmental soil condition (Kwasniewski et al., 2016).

Communication between aerial and belowground organs seems to underlie heat tolerance and root response in some crops. Several studies made with tomato have shown that the more heat tolerant varieties are those that have a higher root activity or a larger RSA. Wider root system can access to more water and nutrient sources, increasing the water uptake and letting the leaves to increase its evapotranspiration rate, cooling their canopy temperature and improving the photosynthetic rate. This in turn allows that larger quantity of assimilates can be used to boost root growth (Shaheen et al., 2016; Zhou et al., 2019). On the other hand, it has also been observed that carbon translocation from shoots to roots is inhibited at high soil temperatures. Under high temperature field conditions, wheat root growth is diminished due to a reduction in the carbon partitioned belowground, and the number, length and diameter of roots are especially affected (Batts et al., 1998). Similarly, in grape, an increase in the temperature reduces root growth rate whereas shoot growth increases due to alteration of assimilate partition (Mahmud et al., 2018). This sink effect of the aerial part of the plants is mostly observed during the reproductive stage, when the carbon partitioning to the root decreases to help flowering and seed development. In summary, warmer soils cause alteration in RSA and root functionality triggering numerous changes in the whole plant in order to adapt to this climatic variance.

One more aspect of root adaptation that is being increasingly explored is the effect of gradient temperature on root architecture. As soil warming reduces downward, progressively deeper soil layers become better suitable for root growth affecting differentially the upper and lower part of roots (Parts et al., 2019). Thus, roots of barley seedlings exposed to uniform temperature or to a vertical gradient respond with significant differences in terms of biomass production and root architecture (Füllner et al., 2012). Other soil conditions associated with soil temperature that also differentially affect root architecture are soil compaction, nutrient composition and moisture. To respond to all these heterogeneous soil environments, crops produce compensatory effects regarding root system architecture and root growth dynamics. In order to capture the best root ideotypes, successful root mechanisms need to be identified by deep phenotyping in complex soil environments and climates. Ideally these ideotypes not only have to respond to specific growth locations but to different dynamics of the stress since increasing temperatures are to be expected as short heat waves or increase seasonal mean temperatures. Finally, farming practices, including plant density, and water and fertilization regimes that directly impact on root development could be crucial to mitigate the unfavorable effects on roots of higher soil temperatures (Pfeifer et al., 2014; Hecht et al., 2016). In this context, modeling of the root behavior under different scenarios including genotype, environment and management will be need to test root traits value for breeding new varieties adapted to increased temperatures.

### Hormonal Response

Several plant hormones that take part in root development and growth have been described to mediate temperature stress response in this organ. In particular, a role of BRs (Bajguz and Hayat, 2009; Anwar et al., 2018), salycilic acid (SA) (Dat et al., 1998), ethylene (ET) (Lin et al., 2009), abscisic acid (ABA) and cytokinin (CK) (Vishwakarma et al., 2017) has been reported in several crops. Temperature-mediated alteration of these hormone levels trigger signal transduction pathways that prepare plants to overcome the stress situation. Key phytohormones including ABA, SA, and ET increase their levels under heat stress, while others such as CK, auxin (AUX), and gibberellins (GAs), decrease (Talanova et al., 2003; Larkindale and Huang, 2004; Larkindale et al., 2005; Nolan et al., 2017, 2019).

Regulation of root response to temperature is mediated by BRs signaling in Arabidopsis. Increased growth temperature reduces the level of the BR receptor BRI1 to downregulate BR signaling and increases root elongation independently of auxin (Martins et al., 2017). Interestingly, it has been proposed that downregulation of BR signaling by temperature elevation could promote GA-dependent root growth. In contrast, in crops, different behavior of BRs has been reported. The application of 24-epibrassinolid (24-EBR), a functional BR, to tomato and oilseed rape seedlings inhibits root elongation in both species but increase their acquired thermotolerance. Molecular analyses of 24-EBR treated and untreated seedlings show that this thermotolerance is a result of increased levels of HSPs (Dhaubhadel et al., 1999, 2002). On the contrary, transgenic lines of oilseed rape overexpressing *AtDWF4*, an Arabidopsis gene encoding an enzyme that catalyzes a bottleneck step in BR biosynthesis, shows an increased root length and fresh and dry root weight. However, the transgenic plants show an increased thermotolerance, and consistent with the results in tomato and oilseed rape, the level of different HSPs gene family members were increased (Sahni et al., 2016).

Improved plant tolerance to heat stress mediated by SA has also been reported in crops (Khan et al., 2015; Nazar et al., 2017). In soybean, wheat, maize and chamomile, this tolerance seems to be mediated by the growth-stimulating effects of SA (Rivas-San Vicente and Plasencia, 2011). Additionally, exogenous SA has a protective role in mitigating extreme temperature-induced damages in different crops (Hasanuzzaman et al., 2017). In grape cultivars root-derived SA have a role in the response to aboveground high temperature. The increase in temperature did not affect free SA content in roots but reduced the levels of conjugated SA, a storage form of this hormone. It is proposed that the sensing of warmer temperatures causes roots to send its conjugated SA reserves to the aboveground parts of the plant where is transformed into free SA to promote the adaptation and resistance to heat stress (Liu et al., 2008).

ET also takes part in root adaptation to increased temperatures. ET production is increased under heat stress, although exogenous ET application cannot confer heat tolerance (Müller and Munné-Bosch, 2015). Nevertheless, thermotolerance is enhanced in rice seedlings under heat stress by an increase in the levels of ET (Wu and Yang, 2019). In sorghum, heat induced inhibition of root elongation and cell production rate is affected by ET levels (Prasad et al., 2008). Likewise, in lettuce, temperature promotes the synthesis of ET. Moreover, exogenous ET application to the root causes heat stress symptoms including reduced root length and surface area and increased root diameter. Application of ET biosynthesis inhibitors to plants exposed to heat alleviates the root growth inhibition. Interestingly, ET effect in this crop has been linked to a similar root-to-shoot communication mechanisms described for SA signaling. Higher ET biosynthesis produced by increased temperatures causes an efflux of ACC, the ET precursor, to the shoot via xylem. ACC then promotes thermotolerance in aboveground tissues by the reduction of oxidative damage and maintenance of chlorophyll content (Qin et al., 2007).

ABA is one of the main hormones to control tolerance to abiotic stress and its biosynthesis is promoted by these stresses also in roots. In cucumber, the application of higher temperature to the whole seedling increases the levels of ABA in both leaves and roots (Talanova et al., 2003). ABA seems to improve heat tolerance through exogenous application or by manipulation of ABA-related genes in some crops. This tolerance is achieved by increasing leaf photochemical efficiency and membrane stability or by induction of HSF (Abass and Rajashekar, 1991; Zhou et al., 2014; Wang et al., 2017). ABA also seems to increase root hydraulic conductance and promote root hair development during adverse environmental situations (Vishwakarma et al., 2017) and it has been suggested as a potential candidate of root-to-shoot communication (Talanova et al., 2003).

CKs are one of the key regulators of root system architecture and they have been implicated in heat stress. In contrast to their role in promoting growth in the shoot, CKs reduce root growth, by inhibiting primary root elongation and promoting cell differentiation in the root apical meristem (Dello Ioio et al., 2008). They are also regulators of root branching (Chang et al., 2015). A decrease in CK levels or a reduction in CK signaling can lead to an enlarged root system improving temperature root response (Bielach et al., 2017; Kieber and Schaller, 2018). Contrarily, stress driven alteration of *CKX1* levels in roots, a CK oxidase/dehydrogenase (CKX) enzyme that regulates CK degradation, results in enhanced drought and heat tolerance in tobacco. The enhanced stress tolerance of these plants has been correlated with raised bioactive CK levels during the early phase of the stress response (Macková et al., 2013).

In summary, several hormones are known to control root growth and are in charge of controlling this process during high temperature stress. Modulation of hormonal signaling in roots in response to heat not only prepares this belowground organ to respond to this stress but also the whole plant since some hormones like SA, ET and ABA could act as intercommunication signals between the root and the aboveground organs.

### Metabolic Response

During heat stress, plant roots suffer large quantity of metabolic changes to maintain homeostasis and allow the plant to survive. It has been suggested that overall alteration of metabolic pathways probably depend on the sensitivity to high temperature of key metabolic regulatory enzymes. Different studies carried out in crops and fodder species shows a common pattern in the response of primary and secondary metabolism to heat stress in roots. Main carbohydrates such as glucose, fructose, galactose, sucrose or xylose are usually lower after the root experience high temperatures, as well as the levels of several glycolytic cycle enzymes (Ribeiro et al., 2014; Aidoo et al., 2016; Sun et al., 2016). In, cassava, warmer soils inhibit starch biosynthesis through the direct decrease of enzymatic activity or down regulation of transcriptional levels of the main starch biosynthesis enzyme (Ma et al., 2018). Other sugars and polyols such as raffinose, galactinol, and glycerol that has been described as stress tolerance compounds increase its content during stress conditions (ElSayed et al., 2014; Salvi et al., 2018). In contraposition of down-accumulation of carbohydrates, some amino acids seem to be accumulated during heat stress. This negative correlation between sugars and amino acid appears to be provoked by the inhibition of carbon assimilates supply to the roots during heat stress. One of the accumulated amino acid is proline, an osmoprotective compound, used to avoid molecular and cellular damage during stress situations (Szabados and Savouré, 2010). Increase temperature also regulates significantly lipid metabolism probably associated to the cell membrane rigidity needed to counteract the fluidity provoked by warmer soils. Thus, fatty acids, phospholipids and glycerolipids shows a reduction in their accumulation after exposing the plant to heat stress together with TCA cycle intermediaries and related enzymes (Ribeiro et al., 2014).

There is fewer and fragmentary data concerning secondary metabolism response to rising temperatures in roots. In maize, increase in temperature causes a decrease in the level of secondary metabolism compounds such as fitosterols and terpenoids (Sun et al., 2016), but in castor bean, although β-sitosterol levels decrease, campesterol storage is increased. The levels of other metabolites like tocopherol, squalene and ricinine, also change in response to heat.

During heat stress, as with other stresses, the intracellular levels of ROS increase sharply. Although it could act as a signaling molecule, higher levels of ROS cause damage at cellular level and interfere with protein and enzymatic activities and gene expression. It has been reported in several crops that the high temperatures promote the expression of ROS scavenging enzymes such as catalases (CAT), peroxidases, superoxide dismutase (SOD) and ascorbate peroxidase to counteract ROS damage (Gill and Tuteja, 2010). Glutathione (GSH) has been described to take part in thermotolerance in eukaryotic organisms by scavenging ROS (Colville and Kranner, 2010). Under heat stress, roots use cysteine to synthesize GSH that could increase the thermotolerance of these organs (Nieto-Sotelo and Ho, 1986). NO and H_2_S are two gaseous molecules that act as signaling compounds during different developmental processes, including root morphogenesis, and stress situations, like heat stress. It has been described for both molecules that its external application confers thermotolerance in both shoot and roots (Li et al., 2013; Singh et al., 2019).

Altogether, significant changes in metabolism in response to high temperature have been reported in different crops directed to alleviate the damage triggered by this stress. Although significant information in this process has been conveyed from several groups, the complete picture of how temperature regulates metabolism in roots is far from been complete. A substantial effort in the study of this regulation will be needed to understand how metabolic changes are integrated in the overall response of roots to this stress.

### Genetic and Molecular Regulatory Pathways

High temperature triggers significant molecular changes in plants, including global transcriptomic reprogramming and changes in protein profiles, to adjust plant growth to this stressing environmental situation. A large number of transcripts and proteins alter their expression and levels in response to heat stress in roots. From these changes, a pattern of stress response reflecting the physiological, morphological and hormonal changes that we have previously described could be drawn. Thus, most of the differential transcripts and proteins represent genes that are involved in primary and secondary metabolism, such as genes related to ROS scavenging, as SOD or CAT and GSH synthesis to sugar and flavonoid biosynthesis; from calcium and signal transduction kinases to proteins related with the regulatory pathways of several hormones (such as ET, SA, JA, ABA, and CK); or from lipid signaling to heat shock proteins and factors (Bita and Gerats, 2013; Valdés-López et al., 2016; Jia et al., 2017; Carrera et al., 2018; Wang et al., 2018a, b).

Activation of *HSPs* and *HSFs* gene families seems to be a universal response to high temperature being found in all organisms from humans to plants. Consequently, several of these genes encoded proteins have been associated to thermotolerance in different crops. In wheat, *HsfA6f* overexpression enhances thermotolerance through the induction of several *HSP* and heat responsive genes. It also activates raffinose and galactinol biosynthesis enzymes and ROS scavenging enzymes by binding to the heat shock elements in the promoters of these genes (Xue et al., 2015). In many plant species, response to heat stress is particularly dependent upon induction of *HSP70* and *HSP101* (Queitsch et al., 2000). In maize, HSP101 regulates root elongation in both normal conditions and mild-heat stress and is needed during germination to balance growth and tolerance establishment (Nieto-Sotelo et al., 2002). Interestingly, it has been observed that differences in thermotolerance between rice cultivars could be mediated also by differences in HSP101 and HSA32 protein levels (Lin et al., 2014). Similarly, in pepper cultivars, HSP25.9, a HSP20, could also be mediating thermoresponse by reducing the accumulation of ROS, enhancing the activity of antioxidant enzymes and regulating the expression of stress-related genes (Feng X. H. et al., 2019).

Heat response encompasses different regulatory gene networks involving specific set of transcription factors, protein kinases and other signaling related proteins (Ohama et al., 2017). In several crops, specific families of transcription factors are candidates to mediate heat stress response in roots. Thus, HD-ZIP and NAC transcription factors are induced by heat stress in potato and radish (Karanja et al., 2017; Li et al., 2019). In batata, ABF4, an ABA-responsive element binding factor that is up-regulated under heat stress promotes the expression of several stress responsive genes and mediate root elongation response (Wang W. et al., 2019). In rice, ZFP350, a Zinc Finger Protein (ZFP) transcriptional factor, is specifically expressed in roots and up regulated by heat. ZFP350 seems to control root response to high temperatures by promoting the expression of stress responsive genes like *HSP70* (Kang et al., 2019). In tomato, a GRF transcription factor, GRF6, is regulated by several stresses including heat through a hormonal mediated pathway (Khatun et al., 2017). Another group of important regulatory proteins that are induced after heat sensing are diverse kinases such as CDPKs or MAPKs (Wang et al., 2018a). A putative rice orthologue of Brassinosteroid insensitive 2 (BIN2), a glycogen synthase kinase3-like gene 1 (GSK1), that acts as repressor of BR signaling seems to mediate heat tolerance in roots (Koh et al., 2007). In pepper, WAKL20, a wall associated RLK-like (WAKL) kinases acts as a negative regulator of thermotolerance by down regulating ABA–responsive genes that in turn decrease plant ABA sensitivity during root growth (Wang H. et al., 2019). Other signaling pathways involving hormone responses are those related with Proline Rich Proteins (PRPs). This family of proteins has been described in several crops as part of root developmental and stress response processes. RCc3, a rice root specific PRP, improves RSA during heat stress by promoting auxin efflux, biosynthesis and accumulation in the roots (Li et al., 2018).

Stress response mediated by increased temperatures also alters several proteins levels through post-translational modifications (Ahmad et al., 2016; Wu et al., 2016; Kosová et al., 2018). These post-translational modifications included phosphorylation, sumoylation or ubiquitination events. For example, the differential phosphorylation levels of two isoforms of fructose-biphosphate aldolase seems to underlie the contrasting heat tolerance in roots of two C3 grass Agrostis species, *A. scabra* and *A. stolonifera* (Xu and Huang, 2008). Also sumoylation levels are altered in several crop roots under heat stress pointing to this protein modification as part of the root response to high temperatures (Augustine et al., 2016; Li X. et al., 2017). Finally, in tomato, ShATL78L, a RING finger protein, enhances multiple abiotic stresses tolerance, including heat, by interacting with a subunit of COP9 signalosome complex and therefore altering ubiquitin-mediated protein degradation (Song et al., 2016).

In recent years, several epigenetic and chromatin-based mechanisms have been implicated in the regulation of heat responsive genes and their function but few examples have been described in crop roots. These epigenetic mechanisms include DNA methylation, histone modifications, histone variants such as the previously mentioned H2A.Z variant, small RNAs and miRNA (Kim et al., 2015; Liu et al., 2015; Lämke and Bäurle, 2017; Saraswat et al., 2017). In rice, several microRNAs show a differential expression in roots of contrasting heat response cultivars. Similarly, in barley, a heat-induced increase in *miR160a*, down-regulates the expression levels of *ARF17* and *ARF13*, which could affect shoot morphology and root growth (Kruszka et al., 2014). In maize roots, the expression and acetylation levels (histone 3 lysine residue 9, H3K9; and histone 4 lysine residue 5, H4K5) of two genes related to lateral root development (*HO1* and *GSL1*), are decreased under heat stress suggesting a mechanism mediated by up-regulation of histone acetyltransferases (HATs) in the root response to this stress (Zhang et al., 2018).

In summary as we have described briefly, there are an increasing number of regulatory mechanisms that are being implicated in the control of heat response in root of different crops. Although there are still many gaps in our knowledge of how all these mechanisms work, all this mounting information will be crucial to expand the set of molecular targets that could be used to improve heat tolerance in crops.

### Increased Temperature Associated Root Traits

Breeding of new cultivars able to overcome the challenging new environmental conditions driven by climate change must incorporate traits regarding root architecture (Koevoets et al., 2016). The potential of roots to boost crop productivity has been establish in several studies where correlations between root traits and yield have been determined (Bray and Topp, 2018; Robinson et al., 2018; Jia et al., 2019). This close relation is confirmed by the co-ocurrence of QTLs for root traits and grain yield and other agronomic traits associated to productivity in different crops (Maccaferri et al., 2016; Ju et al., 2018). Root traits like deep rooting or root angle seem to increase vegetative growth and subsequent grain filling but are also context dependent. Deep root systems developed in limiting water conditions increase grain yield by providing access to residual water in deeper soil layers (El Hassouni et al., 2018). Additionally, root length has been correlated with flowering traits in different crops but how this association takes place is not well known (Voss-Fels et al., 2018). Similarly, several above-ground traits are influenced by root behavior under different stress conditions including high soil temperatures (Batts et al., 1998; Arai-Sanoh et al., 2010). All these studies highlight the idea that a complete plant phenology has to be taken into account when root traits are selected for breeding for adaptation to avoid yield penalties.

As we have seen, roots are very plastic to environmental conditions and display a large range of highly variable physiological and morphological traits to adapt root architecture and functionality to disadvantageous conditions. Classical breeding trials were designed to select for cultivars with high yield using non-limiting nutrients and non-challenging environmental conditions which has often led to selection for smaller and less plastic roots (White et al., 2013). Moreover, modern cultivars have relied on the monitoring and selection of above-ground traits looking for increasing biomass into the shoots rather than into the roots, that it turns has selected for smaller root sizes and root:shoot ratios (Waines and Ehdaie, 2007; Friedli et al., 2019). As a result, root traits have been usually downplayed in breeding programs but numerous studies have shown the correlation of root traits with enhanced tolerance and productivity in different crops species (Den Herder et al., 2010). These studies highlight the potentiality of root traits as tools for breeding high tolerant crops (de Dorlodot et al., 2007). Heat stress tolerance as other abiotic tolerance seems to be a multigenic trait and the candidate genes are poorly known. Root traits are genetically complex and more difficult to measure (Wasaya et al., 2018). Everything considered, improving this stress tolerance in root crops is a very limiting step in plant breeding. Roots are challenging to evaluate in the soil and this has been a major reason for the poor attention that they have been paid in breeding programs in the past. Numerous methods of phenotyping have been used, from laboratory-based methods including the use of soil-free media pots, rhizoboxes, hydroponics or semi-hydroponics media combined with high-throughput digital phenotyping or 3D imaging systems (Walter et al., 2015; Voss-Fels et al., 2018; Jia et al., 2019; Ma et al., 2019; Qiao et al., 2019) to field shovelomics (Trachsel et al., 2011). But still all these methods are generally expensive or/and time-consuming, so better and affordable tools to improve analysis of root traits are still needed. Nevertheless, significant information of root adaptation to changes in temperature has been provided by exploiting genetic variation associated to root traits.

Genome-wide association studies (GWAS) have been widely used during the last few years to identify loci on tolerance to extreme temperatures in crops (Hu et al., 2017; Maulana et al., 2018; Jamil et al., 2019; Jia et al., 2019; Oladzad et al., 2019) or root architecture (Li X. et al., 2017; Li Y. et al., 2017) but analyses focused on root response to temperature are still lacking. Similarly, QTL mapping has been used to narrow down regions of crop genomes related to root architecture (Gong et al., 2015). Although several studies has identified, mapped and predicted potential genes candidates for QTLs associated with heat or high temperature tolerance in several crops like tomato (Wen et al., 2019), maize (Van Inghelandt et al., 2019), barley (Arifuzzaman et al., 2014) and wheat (Sharma et al., 2017), very few have been focused on root related traits. Thus, in wheat, QTLs for cooler canopy temperature (QTL-CT) are associated to a higher number of superficial roots compared to deep roots (Pinto and Reynolds, 2015). QTL analyses also in wheat show a coincidence of a QTL for heat and drought tolerance suggesting a common genetic basis for adaptation to both stresses. This QTL seems to be associated with changes in root distribution to increase water availability (Pinto et al., 2010). Likewise, a later analysis in wheat to identify meta-QTL associated with adaptation to drought and heat stress, shows that a large number of QTLs are shared to both heat and drought response and two of them are associated to higher root length (Acuña-Galindo et al., 2015). Similarly, in rice, studies with recombinant inbred lines (RILs) obtained from crosses between heat tolerant and non-tolerant cultivars have identified QTLs associated with root length under heat stress (Ps et al., 2017; Kilasi et al., 2018), and in barley, two heat-stress QTLs are adjacent to a QTL reported for root length and root-shoot ratio (Gous et al., 2016). In maize, association mapping studies between inbred lines with different heat tolerance show a significant effect on lateral and axillary root elongation rates in these genotypes (Trachsel et al., 2010; Reimer et al., 2013). Interestingly, this change on root architecture coincides with the proposed maize ideotype for the root system which represents steep and deep roots, and reduction of the metabolic cost of soil exploration (Lynch, 2013; Gong et al., 2015). Altogether these analyses reinforce the idea that better developed roots help the plant to increase the water intake during heat stress that in turn increases the evapotranspiration rate and decreases the aboveground temperature allowing a better photosynthetic ratio and crop yield. However, the optimal RSA could be different in each targeted environment and breeding efforts have to account for these differences. Moreover, some of the adaptive root traits are only conveyed when roots are under specific stresses making phenotyping and evaluation of root traits even more challenging (Alahmad et al., 2019). Thus, drought induced deep rooting that reduces root growth in upper soil layers compare to shallow roots is an effective strategy when heat is combined with low moisture soil but has yield penalties in moisture rich soils (Comas et al., 2013; El Hassouni et al., 2018). Combination of context dependant or independent root traits has been proposed as solution for adaptation to target multiple environments. For that purpose, analysis of natural variation and wild relatives have been used to uncover some of the processes underlying either root growth or responses to temperature changes. New root trait alleles would be uncovered using this strategy but the effectiveness of these tools to analyze root response to increase temperature in crops is yet to be explored (Ristova and Busch, 2014; Blackman, 2017; Driedonks et al., 2018; Ristova et al., 2018; Wang et al., 2018c).

In summary, the information gathered from all these studies has been very useful to shed light onto some of the possible strategies adopted by the roots to confront temperature stress. These strategies include primarily alteration of RSA and adjustments of their interchange with aboveground organs. However, there are still many other avenues to extensively exploit the plasticity of the roots. In modern agricultural system, crops are highly densely planted and root traits related with root angle or root occupancy could be highly valuable (Meister et al., 2014; Hecht et al., 2016). In cereals, with a root system that changes during their lifespan (postembryonic root are different from embryonic roots), a multi-trait approach considering all root types will be needed to uncover useful genotypes. Lastly, root traits identifyed on multi environmental field trials considering complex and concomitant soil conditions seems a very promising approach to adapt root system of crops to climate change.

## Root Responses to Temperature Associated Abiotic and Biotic Stress

**FIGURE 4:**
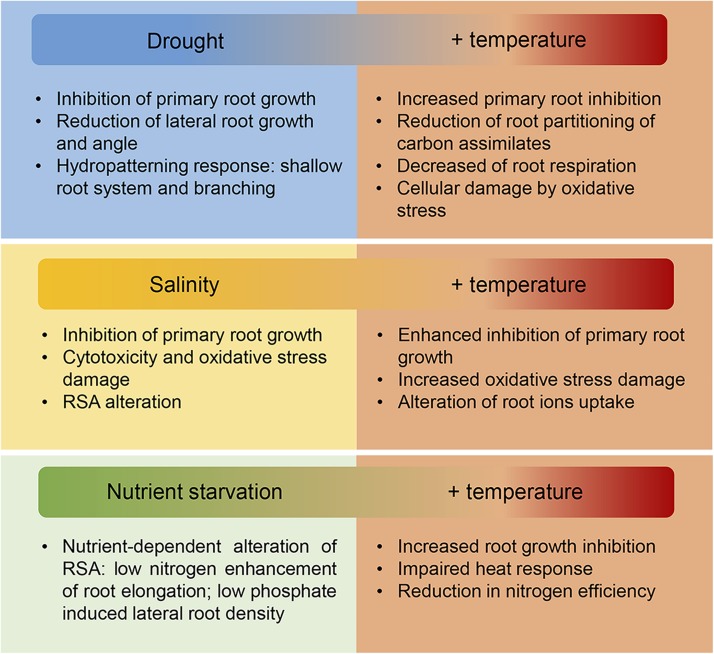
Effect of increasing temperature and associated abiotic stresses on root growth. In the field, the increase in temperatures driven by climate change is normally accompanied by water deprivation provoked by enhanced evapotranspiration of the soil and plants. Moreover, increased soil salinization and changes in the nutrient composition of the soil further compromise plant growth. Roots are essential for water, ions and nutrient uptake therefore the adverse effects on roots of these combined stresses as is summarized in this figure, directly affects crop productivity on the field. New crops with improved root response to a variety of biotic and biotic stresses will be needed to maintain yield stability under the changeable environmental conditions driven by climate change.

In field conditions, under the predicted climate change scenario, the increase in temperatures is usually accompanied by an enhanced evapotranspiration of soil and plants following by an increase in drought incidence and soil salinization. Additionally, higher temperatures could lead to an increased virulence and expansion of crop pathogens (Mahalingam, 2015). Therefore, in order to improve root adaptation in crops we need to consider how combined stress responses affect root growth (Figure 4; Koevoets et al., 2016).

### Abiotic Stresses

Water is one of the most limiting factors for crop growth and its availability is determined by weather, soil structure and root uptake. Root growth response to water deprivation usually includes inhibition of lateral root growth and enhancement of primary and secondary root growth. But when scarcity of water is more severe a drought avoidance program is deployed to direct root growth and branching into regions of soil where these resources are more abundant (Dinneny, 2019). ABA and auxins regulate this hydropatterning response (Orosa-Puente et al., 2018). Interestingly, a major rice QTL for the control of deep rooting, *DRO1*, modulates yield under drought stress by affecting root growth angle (Uga et al., 2013). Severe drought conditions, in addition to higher temperatures, provoke a strong inhibition on root respiration rate and growth as well as a reduction in the partitioning of carbon assimilates to the roots (Prasad et al., 2008). The root response to the combined effect of heat and drought could vary depending on the crop and the developmental stage. Thus, root growth seems to be directly affected by water deficit and temperature to a greater extent in C3 than C4 crops. Sunflower, a C3 plant, responds to the combined stress situation by partitioning carbon assimilates to the root to promote growth and ensure water availability. In maize, a C4 plant, increased temperature inhibits root elongation (Killi et al., 2017). In barley, plants at heading stage seem to be more sensitive to both stresses than plants during vegetative growth, and plants that show greater carbon assimilates partitioning to the root during heading also show lower yield and lower quality traits (Mahalingam and Bregitzer, 2019). In tomato, heat stress causes an increase in root activity that is translated into an increase in water uptake. But this response is reversed when this stress is combined with drought. In addition, the combination of both stresses accelerates the harmful effects of each stress (Zhou et al., 2019). At cellular level, the combination of heat and drought causes oxidative stress (Zandalinas et al., 2018). Roots exposed to these conditions accumulate more proline and increased expression of antioxidant enzymes to suppress the potential molecular damage (Selote and Khanna-Chopra, 2010; Sekmen et al., 2014). Lastly, interesting information could be deducted from the ability of some plants such as members of the *Cactaceae* family to grow in arid desert that combine both stresses. Root traits from these plants includes the iterative senescence of the primary root tip, which facilitates rapid branching and shallow root system growth during the rare precipitation events occurring in the desert (Shishkova et al., 2013).

Soil salinization is a major threat that negatively affects crop productivity. Salinity impairs plant growth and development *via* water stress and cytotoxicity due to excessive uptake of ions such as sodium (Na^+^). Additionally, salinity is typically accompanied by oxidative stress due to generation of ROS (Isayenkov and Maathuis, 2019). Contrary to what happen with heat, roots are more resistant to salt stress than leaves, but this stress still severely inhibits root growth and provokes damages and alterations in the RSA (Robin et al., 2016). These alterations seem to depend on the crop. Thus, in wheat, root elongation is promoted by the combination of heat and drought but high salinity alone inhibits root growth. Furthermore, when plants were treated with salt and heat, the inhibition caused by the salinity was stronger (Keleş and Öncel, 2002). Similarly, in barley, root growth is severely inhibited and ROS levels sharply increase. To counteract this response, plants accumulate a great quantity of proline and other osmoprotectants, and increase the expression and activity of ROS scavenging enzymes. SA may have a role in this tolerance process by promoting the biosynthesis of osmoprotectants and regulating the activity of several ROS scavenging enzymes (Torun, 2019). On the contrary, in tomato, heat seems to alleviate salinity damage by increasing evapotranspiration and photosynthetic rates. The combination of both stresses also seems to alter the uptake, transport and accumulation of Na^+^ and K^+^. So, under heat and salinity stresses, tomato accumulates Na^+^ ions in the root in order to decrease the level of this ion in leaves and evict photosynthesis alteration (Rivero et al., 2014).

Nitrogen levels in soil also affect root viability, thereby higher or lower nitrogen levels than optimum negatively alters root growth. Additionally, proper N availability is important for plant resistance to stress conditions. In warm soils during spring, roots have to mobilize nitrogen reserves to respond to increased plant growth demand including enhanced root growth. Therefore, it has been suggested that supplying N to the soil could mitigate the effect of temperature on root growth in a similar way (Waraich et al., 2012). Application of nutrients like N, K, Ca, and Mg seems to reduce the toxicity of ROS whereas K and Ca improve intake of water and help to maintain high tissue water potential. One challenge to enhance nitrogen efficiency in crops is to understand how lroots respond to low nitrogen and how the modulation of root architecture is coordinated to maximize nutrient acquisition in variable ambient temperatures. Positive or negative coincidences between N uptake and heat tolerance have been observed in different species (Yan et al., 2012; Giri et al., 2017). Thus, N availability influences HSP levels in maize (Heckathorn et al., 1996) and in the perennial grass, *Agrostis stolonifera*. Combination of nutrient deficiency with higher temperatures in soils, further alters HSP synthesis (Wang et al., 2014).

A major constrain for crop productivity is the deficiency of resources, water and nutrients, in the soil surrounding the root system. As we have seen, roots alter their physiology and morphological traits to increase their efficiency when it is compromised by environmental conditions as increased temperature or a combination of stresses. This root multi-adaptive response need to be incorporated in the breeding of new cultivars to increase their adaptation to unstable climates.

### Biotic Stress

Environmental conditions profoundly affect plant disease development; however, the underlying molecular bases are not fully understood. Weather plays a large role in determining the outcome of plant–pathogen interactions, and disease epidemics are more likely to occur when environmental conditions are detrimental for the plant. For example, it is known that temperature fluctuation is a key determinant for microbial invasion and host evasion. Thus, there is an observed pattern of movements driven by global warming effects on crop pathogens and pests, and/or on the availability of crops to cope with them (Bebber et al., 2013). Other outcomes of warming temperatures are that new pathogen strains better adapted to these temperatures may become prevalent and the rise of more aggressive plant disease vectors (Velásquez et al., 2018). High temperature enhances plant disease susceptibility, attenuating disease resistance and promoting pathogen growth (Fujita et al., 2006; Huot et al., 2017). Several mechanisms seem to be implicated in this effect. Increase in temperature causes a decrease in the elicitor detection by the plant and the breakdown of effector-triggered immunity (de Jong et al., 2002; Alcázar and Parker, 2011; Cheng et al., 2013; Hua, 2013). Examples of this effect in roots have been already described. Changes in weather conditions including increased mean winter temperatures have favored infection by several *Phytophthora* spp. species that are responsible for increasing amounts of root rot in forest trees (Jung and Burgess, 2009; Sturrock et al., 2011). Additionally, other soil-borne root diseases seem to be more severe under increased temperature conditions (Elad and Pertot, 2014). Plant response to pathogens and adverse environmental conditions is challenging. Since both responses share many components, plants need to trigger a balanced response between the tolerance and defense response. In fact, mounting evidence suggests that hormone signaling pathways regulated by ABA, SA, JA and ET, as well as MAP-kinase cascades and ROS signaling pathways, play key roles in the crosstalk between biotic and abiotic stress signaling such as heat (Chen et al., 2015; Zhai et al., 2017). In this context, stress caused by temperature has been shown to negatively affect the plant ability to respond to pathogens through changes in ABA levels that influence defense responses involving SA, JA, or ET (Asselbergh et al., 2008). An emerging field in abiotic and biotic interaction is that involving plant–microbiome interaction. Disease-suppressive soils with enrichment in specific bacterial clades are able to protect against soil-borne pathogens including fungal root pathogens (Mendes et al., 2011; Philippot et al., 2013). But the alteration of the microbiome and the reduction in number and diversity caused by higher soil temperature could lead to the loss of pathogen suppression capacity of the rhizobiome (Mendes et al., 2011; van der Voort et al., 2016). Although much work is still to be made to understand the crosstalk between environmental conditions such temperature and pathogen interaction in plants, there is an urgency to produce disease-resistant crop plants that are resilient to climate change.

Crop breeding programs are incorporating the response to a combination of different stresses in the evaluation of new varieties. This type of analysis although challenging due to the requirement of multi-environment field trials, are becoming a necessary requisite to assess the real value of the traits to be integrated in the varieties. This is especially relevant in the context of root traits given the high plasticity of the RSA to changes in the environmental conditions and composition of the soil. Root traits aimed to improve the stability of crop productivity have to be able to respond favorably in all the environmental contexts.

## Challenges and Future Solutions

Humanity’s main challenge of this century is to feed the growing population in a context of climate change. Between 2030 and 2050 the population will have increased to 9,000 million people whereas global temperature will have increased between 1.5 and 2°C. The alteration of climate and the more common appearance of extreme events, in addition to higher temperatures, will negatively affect crop yield. Global food security would be endangered resulting in the increase of food prices and food shortages, and in consequence increasing global hunger, poverty and inequality. So, it is of paramount importance the improvement of crop tolerance to abiotic and biotic stresses in order to confront climate change effects.

**FIGURE 5:**
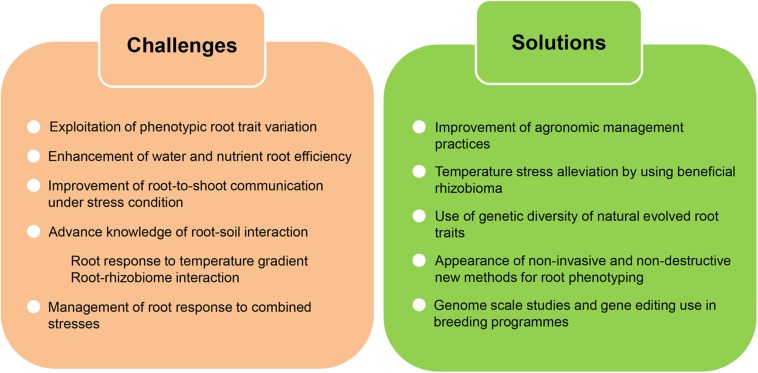
Challenges and potential solutions to improve crop root adaptation to climate change. Global alteration of climate in addition to higher temperatures will negatively affect crop yield. There is an urgent need to improve and maintain crop productivity under these climatic constrains and root traits withhold the potential to reach this goal. In order to confront climate change effects we still have to overcome a few challenges, largely concerning the necessity to increase our knowledge of different aspects of the root adaptation process. New solutions bringing together technical and conceptual advances in the analysis of root traits will drive this advancement.

Root traits still withhold the potential to reach this goal, but first the extensive existing phenotypic variation in these traits must be studied and analyzed (Figure 5). Moreover, the improvement of root capability might help to mitigate the harmful effect of agriculture on environment. Better root performance could reduce the water used for irrigation during heat waves or the massive fertilization of fields. On the other hand, root development and capacity should be improved without sacrificing other traits regarding aboveground development or yield. How temperature related changes in root architecture might affect the aerial part of the plant is not well understood and in particular, the signaling from the root to the shoot (or vice versa) in order to prepare the whole plant for the heat stress. Having a better comprehension of the genetic and molecular regulatory pathways underlying root-to-shoot interaction under stress condition could be useful to improve root performance without altering shoot development related traits.

Another challenging aspect to consider is that the temperature of the soil is not uniform, but it maintains a gradient that decreases with depth. This gradient varies depending on the soil composition, a factor contributing to heat conduction and convection. Consequently, the temperature of the soil, and the root, decreases with depth increasing the complexity of root response to heat and its study. The complex relation between the root and the soil increases even more when the role of the rhizosphere is added to the analysis. The potential effect of the rhizosphere to defend or prepare the plant against biotic and abiotic stresses is little explored. Unraveling the complex interaction between the rhizobiome and the root, during heat stress at a molecular and cellular level is essential to understand whole-plant heat tolerance processes. As we have seen throughout this review, in the changing climatic condition, the different stresses do not occur separately but very often they appear together. How plants response to several stresses simultaneously is a poorly understood process especially in roots compared to the information gathered from aboveground tissues. Better understanding of plant response to each stress or its combination is primary to develop more tolerant crop varieties. In brief, there is still a lot of work to be done to obtain potential applications and improvements of root tolerance not only to heat stress but also to other biotic and abiotic stresses.

A first approach to tackle the effect of climate change on crops and at the same time lessen the impact of agriculture is the improvement of agronomic management practices and the use of precise farming. A more efficient use of nitrogen and phosphate fertilizers as well as water could reduce their use in the field. This strategy could help to alleviate the soil deterioration caused by these fertilizers and contribute to reduce water scarcity and pollution. In this context, the optimization of root efficiency in nutrients and water uptake and distribution could lead to a better fertilizer and water management. Better root systems provided by cover crops could be useful in managing and preserve soil quality and soil moisture. Moreover, the use of leguminous plants as cover crops could also be use as a fertilization method due to its symbiotic relationship with nitrogen fixation bacteria. Additionally, the use of better or new agronomic techniques could help to alleviate the increase in soil temperature. For example, no-tillage seems to be beneficial to avoid, or at least, decrease heat stress in root (Wang et al., 2007). Lastly as commented in the previous section, the tailored application of N to the soil could enhance root growth alleviating heat effects.

One of the emerging strategies to approach the use of root traits to fight global warming and its effects on crop yield is the use of the rhizobiome. Plants are able to adjust rhizobiome composition through root exudates that could stimulate the growth of beneficial microorganism in the rhizosphere (Vives-Peris et al., 2018). But changes in soil characteristics lead to a change in root exudates and, in consequence, a change in rhizobiome composition (Philippot et al., 2013). Specific bacteria have been described to enhance plant tolerance to biotic (Santhanam et al., 2015) and abiotic stresses (Rolli et al., 2015). In fact, increased temperature leads to alterations in root exudates that promote some beneficial bacteria that could improve crop survival in this condition (Ali et al., 2011). Harnessing the beneficial interaction between the root and rhizosphere has the potential to improve crop tolerance to various stresses (Ahkami et al., 2017). Moreover, the use of symbiotic or non-symbiotic fungi isolated from plant species that grow in inhospitable environments to provide crops with tolerance to several stresses is also being explored (Singh et al., 2011).

Another focus of attention in the field of root adaptation is the use of temperature adapted wild relatives and landraces. During the domestication of crop species, the main traits selected were those related to a greater yield and quality. In this process the loss of root traits related with stress tolerance probably has happened. Crop wild relatives are a source of genetic diversity of natural evolved root traits including root adaptation to stresses. By analyzing the genome of these plants, the evolutionary pathways taken to gain these traits could be understood and applied in breeding programs. Analyses of crop wild relatives have already shown that their genetic variability is a great field to exploit in breeding programs centred on obtaining new crop varieties with tolerance to diverse stresses (Dempewolf et al., 2010, 2014). For example, wild relatives of pigeon pea (*Cajanus cajan*) and wheat has proven to be a source of genetic resources and traits to improve the tolerance of their related crops to stress conditions (Zaharieva et al., 2001; Khoury et al., 2015; Von Wettberg et al., 2018). In addition to wild relatives, crop landraces are a great source of genetic variability for their adaptation to specific ecosystems and climatic conditions (Cantalapiedra et al., 2017; Carvalho et al., 2017; Sani et al., 2018). On the other hand, latest studies with orphan crops have demonstrated that those crops are a powerful tool to improve their related global-traded crops due to its resistance against unfavorable conditions (Song et al., 2019; Tadele and Bartels, 2019).

Traditionally, one of the main bottlenecks to study root adaptation in crops and wild species has been the technical challenge to phenotype roots as a whole system and in their interaction with the soil. The progressive appearance of non-invasive and non-destructive new methods such as shovelomics, X-ray thomography and magnetic resonance imaging (MRI) to visualize the 3D-configuration of roots is allowing to deepen the study of root development during the whole life cycle of plants (Keyes et al., 2013; Walter et al., 2015). As a result, media that allow direct observation of root development, such as hydroponic culture or the use of gelled media, is being widely used to facilitate these studies. Still more problems arise when the goal is to analyze root soil interaction and specially to emulate soil temperature gradient (Füllner et al., 2012). Different sharing platforms and softwares specifically designed to analyze root traits are easing the study of the root system and the associations of different traits to different stages or root responses (Das et al., 2015; Tracy et al., 2020). Although a few challenges still remain to study root adaptation in crops, new methodologies and tools are constantly being developed. Thus, analysis like the transcriptional landscape of different roots types in wheat (Ramírez-González et al., 2018) or the development of expression tissue profiling similar to eFP browser (Winter et al., 2007) or Tomato Expression Atlas (Fernandez-Pozo et al., 2017) in roots of different crops will be immensely useful.

Once beneficial root traits have been defined and potential gene candidates are identified they must be incorporated into breeding programs. A critical challenge is the time it takes from research finding to implementation in agriculture. Complementary approaches and technologies are needed to accelerate downstream breeding. Between the most promising solutions, crop editing has the greatest potential to improve root performance under various abiotic stresses in relatively short time (Butt et al., 2019). Gene editing driven by tailored strategies focused in specific crop species and stress situation, and a rational design and assembly of appropriated gene combination could result in the generation of new crop varieties able to respond to a particular or a combination of stresses without affecting their yield (Bailey-Serres et al., 2019). This approach, together with powerful genome scale analysis, genome wide association studies and molecular marker assisted breeding are a promiseful alternative to produce new elite varieties adapted to the incoming climatic situation.

## Author Contributions

MP and JC-C wrote the manuscript and draw the figures. LO-S and MB contributed in drafting and revising the manuscript.

## Conflict of Interest

The authors declare that the research was conducted in the absence of any commercial or financial relationships that could be construed as a potential conflict of interest.

## References

[B1] Abass M. Rajashekar C. B. (1991). Characterization of heat injury in grapes using ^1^H nuclear magnetic resonance methods: changes in transverse relaxation times. *Plant Physiol.* 96 957–961. 10.1104/pp.96.3.957 16668281 PMC1080871

[B2] Acuña-Galindo M. A. Mason R. E. Subramanian N. K. Hays D. B. (2015). Meta-analysis of wheat QTL regions associated with adaptation to drought and heat stress. *Crop Sci.* 55 477–492. 10.2135/cropsci2013.11.0793

[B3] Ahkami A. H. Allen White R. Handakumbura P. P. Jansson C. (2017). Rhizosphere engineering: enhancing sustainable plant ecosystem productivity. *Rhizosphere* 3 233–243. 10.1016/j.rhisph.2017.04.012

[B4] Ahmad P. Abdel Latef A. A. H. Rasool S. Akram N. A. Ashraf M. Gucel S. (2016). Role of proteomics in crop stress tolerance. *Front. Plant Sci.* 7:1336. 10.3389/fpls.2016.01336 27660631 PMC5014855

[B5] Aidoo M. K. Bdolach E. Fait A. Lazarovitch N. Rachmilevitch S. (2016). Tolerance to high soil temperature in foxtail millet (*Setaria italica* L.) is related to shoot and root growth and metabolism. *Plant Physiol. Biochem.* 106 73–81. 10.1016/j.plaphy.2016.04.038 27149034

[B6] Ainsworth E. A. (2017). Understanding and improving global crop response to ozone pollution. *Plant J.* 90 886–897. 10.1111/tpj.13298 27739639

[B7] Aksouh N. M. Jacobs B. C. Stoddard F. L. Mailer R. J. (2001). Response of canola to different heat stresses. *Aust. J. Agric. Res.* 52 817–824. 10.1071/AR00120

[B8] Alahmad S. El Hassouni K. Bassi F. M. Dinglasan E. Youssef C. Quarry G. (2019). A major root architecture QTL responding to water limitation in Durum Wheat. *Front. Plant Sci.* 10:436. 10.3389/fpls.2019.00436 31024600 PMC6468307

[B9] Alcázar R. Parker J. E. (2011). The impact of temperature on balancing immune responsiveness and growth in *Arabidopsis*. *Trends Plant Sci.* 16 666–675. 10.1016/j.tplants.2011.09.001 21963982

[B10] Ali S. Z. Sandhya V. Grover M. Linga V. R. Bandi V. (2011). Effect of inoculation with a thermotolerant plant growth promoting *Pseudomonas* putida strain AKMP7 on growth of wheat (*Triticum* spp.) under heat stress. *J. Plant Interact.* 6 239–246. 10.1080/17429145.2010.545147

[B11] Angadi S. V. Cutforth H. W. Miller P. R. McConkey B. G. Entz M. H. Brandt S. A. (2000). Response of three *Brassica* species to high temperature stress during reproductive growth. *Can. J. Plant Sci.* 80 693–701. 10.4141/P99-152

[B12] Anwar A. Liu Y. Dong R. Bai L. Yu X. Li Y. (2018). The physiological and molecular mechanism of brassinosteroid in response to stress: a review. *Biol. Res.* 51:46. 10.1186/s40659-018-0195-2 30419959 PMC6231256

[B13] Arai-Sanoh Y. Ishimaru T. Ohsumi A. Kondo M. (2010). Effects of soil temperature on growth and root function in rice. *Plant Prod. Sci.* 3 235–242. 10.1626/pps.13.235

[B14] Arifuzzaman M. Sayed M. A. Muzammil S. Pillen K. Schumann H. Naz A. A. (2014). Detection and validation of novel QTL for shoot and root traits in barley (*Hordeum vulgare* L.). *Mol. Breed.* 34 1373–1387. 10.1007/s11032-014-0122-3

[B15] Asselbergh B. Achuo A. E. Höfte M. van Gijsegem F. (2008). Abscisic acid deficiency leads to rapid activation of tomato defence responses upon infection with *Erwinia chrysanthemi*. *Mol. Plant Pathol.* 9 11–24. 10.1111/j.1364-3703.2007.00437.x 18705880 PMC6640284

[B16] Asseng S. Ewert F. Martre P. Rötter R. P. Lobell D. B. Cammarano D. (2015). Rising temperatures reduce global wheat production. *Nat. Clim. Chang.* 5 143–147. 10.1038/nclimate2470

[B17] Asseng S. Foster I. Turner N. C. (2011). The impact of temperature variability on wheat yields. *Glob. Chang. Biol.* 17 997–1012. 10.1111/j.1365-2486.2010.02262.x

[B18] Asseng S. Martre P. Maiorano A. Rötter R. P. O’Leary G. J. Fitzgerald G. J. (2019). Climate change impact and adaptation for wheat protein. *Glob. Chang. Biol.* 25 155–173. 10.1111/gcb.14481 30549200

[B19] Augustine R. C. York S. L. Rytz T. C. Vierstra R. D. (2016). Defining the SUMO system in maize: SUMOylation is up-regulated during endosperm development and rapidly induced by stress. *Plant Physiol.* 171 2191–2210. 10.1104/pp.16.00353 27208252 PMC4936565

[B20] Avnery S. Mauzerall D. L. Liu J. Horowitz L. W. (2011). Global crop yield reductions due to surface ozone exposure: 2. *Year* 2030 potential crop production losses and economic damage under two scenarios of O3 pollution. *Atmos. Environ.* 45 2297–2309. 10.1016/j.atmosenv.2011.01.002

[B21] Bailey-Serres J. Parker J. E. Ainsworth E. A. Oldroyd G. E. D. Schroeder J. I. (2019). Genetic strategies for improving crop yields. *Nature* 575 109–118. 10.1038/s41586-019-1679-0 31695205 PMC7024682

[B22] Bajguz A. Hayat S. (2009). Effects of brassinosteroids on the plant responses to environmental stresses. *Plant Physiol. Biochem.* 47 1–8. 10.1016/j.plaphy.2008.10.002 19010688

[B23] Battisti D. S. Naylor R. L. (2009). Historical warnings of future food insecurity with unprecedented seasonal heat. *Science* 323 240–244. 10.1126/science.1164363 19131626

[B24] Batts G. R. Ellis R. H. Morison J. I. L. Nkemka P. N. Gregory P. J. Hadley P. (1998). Yield and partitioning in crops of contrasting cultivars of winter wheat in response to CO2 and temperature in field studies using temperature gradient tunnels. *J. Agric. Sci.* 130 17–27. 10.1017/S0021859697005017

[B25] Bebber D. P. Ramotowski M. A. T. Gurr S. J. (2013). Crop pests and pathogens move polewards in a warming world. *Nat. Clim. Chang.* 3 985–988. 10.1038/nclimate1990

[B26] Bellstaedt J. Trenner J. Lippmann R. Poeschl Y. Zhang X. Friml J. (2019). A mobile auxin signal connects temperature sensing in cotyledons with growth responses in hypocotyls. *Plant Physiol.* 180 757–766. 10.1104/pp.18.01377 31000634 PMC6548272

[B27] Bielach A. Hrtyan M. Tognetti V. B. (2017). Plants under Stress: involvement of Auxin and Cytokinin. *Int. J. Mol. Sci.* 18:1427. 10.3390/ijms18071427 28677656 PMC5535918

[B28] Bita C. E. Gerats T. (2013). Plant tolerance to high temperature in a changing environment: scientific fundamentals and production of heat stress-tolerant crops. *Front. Plant Sci.* 4:273. 10.3389/fpls.2013.00273 23914193 PMC3728475

[B29] Blackman B. K. (2017). Changing responses to changing seasons: natural variation in the plasticity of flowering time. *Plant Physiol.* 173 16–26. 10.1104/pp.16.01683 27872243 PMC5210766

[B30] Boyer J. S. Westgate M. (2004). Grain yields with limited water. *J. Exp. Bot.* 55 2385–2394. 10.1093/jxb/erh219 15286147

[B31] Bravo-F P. Uribe E. G. (1981). Temperature dependence of the concentration kinetics of absorption of phosphate and potassium in corn roots. *Plant Physiol.* 67 815–819. 10.1104/pp.67.4.815 16661760 PMC425778

[B32] Bray A. L. Topp C. N. (2018). The quantitative genetic control of root architecture in maize. *Plant Cell Physiol.* 59 1919–1930. 10.1093/pcp/pcy141 30020530 PMC6178961

[B33] Butt H. Shan-e-AliZaidi S. Hassan N. Mahfouz M. (2019). CRISpr-based directed evolution for crop improvement. *Trends Biotechnol.* 38 236–240. 10.1016/j.tibtech.2019.08.001 31477243

[B34] Cabañero F. J. Martínez V. Carvajal M. (2004). Does calcium determine water uptake under saline conditions in pepper plants, or is it water flux which determines calcium uptake? *Plant Sci.* 166 443–450. 10.1016/j.plantsci.2003.10.010

[B35] Cantalapiedra C. P. García-Pereira M. J. Gracia M. P. Igartua E. Casas A. M. Contreras-Moreira B. (2017). Large differences in gene expression responses to drought and heat stress between elite barley cultivar scarlett and a Spanish landrace. *Front. Plant Sci.* 8:647. 10.3389/fpls.2017.00647 28507554 PMC5410667

[B36] Carrera D. Á Oddsson S. Grossmann J. Trachsel C. Streb S. (2018). Comparative proteomic analysis of plant acclimation to six different long-term environmental changes. *Plant Cell Physiol.* 59 650–650. 10.1093/pcp/pcy029 29444260

[B37] Carvajal M. Cooke D. T. Clarkson D. T. (1996). Plasma membrane fluidity and hydraulic conductance in wheat roots: interactions between root temperature and nitrate or phosphate deprivation. *Plant, Cell Environ.* 19 1110–1114. 10.1111/j.1365-3040.1996.tb00219.x

[B38] Carvalho M. Muñoz-Amatriaín M. Castro I. Lino-Neto T. Matos M. Egea-Cortines M. (2017). Genetic diversity and structure of Iberian peninsula cowpeas compared to world-wide cowpea accessions using high density SNP markers. *BMC Genomics* 18:891. 10.1186/s12864-017-4295-0 29162034 PMC5697113

[B39] Chang L. Ramireddy E. Schmülling T. (2015). Cytokinin as a positional cue regulating lateral root spacing in *Arabidopsis*. *J. Exp. Bot.* 66 4759–4768. 10.1093/jxb/erv252 26019251 PMC4507779

[B40] Che P. Bussell J. D. Zhou W. Estavillo G. M. Pogson B. J. Smith S. M. (2010). Signaling from the endoplasmic reticulum activates brassinosteroid signaling and promotes acclimation to stress in *Arabidopsis*. *Sci. Signal.* 3:1140 10.1126/scisignal.2001140 20876872

[B41] Chen J. Tang L. Shi P. Yang B. Sun T. Cao W. (2017). Effects of short-term high temperature on grain quality and starch granules of rice (*Oryza sativa* L.) at post-anthesis stage. *Protoplasma* 254 935–943. 10.1007/s00709-016-1002-y 27447698

[B42] Chen X. Wang J. Zhu M. Jia H. Liu D. Hao L. (2015). A cotton Raf-like MAP3K gene, GhMAP3K40, mediates reduced tolerance to biotic and abiotic stress in *Nicotiana benthamiana* by negatively regulating growth and development. *Plant Sci.* 240 10–24. 10.1016/j.plantsci.2015.08.012 26475184

[B43] Cheng C. Gao X. Feng B. Sheen J. Shan L. He P. (2013). Plant immune response to pathogens differs with changing temperatures. *Nat. Commun.* 4:3530. 10.1038/ncomms3530 24067909 PMC3901997

[B44] Colville L. Kranner I. (2010). Desiccation tolerant plants as model systems to study redox regulation of protein thiols. *Plant Growth Regul.* 62 241–255. 10.1007/s10725-010-9482-9

[B45] Comas L. H. Becker S. R. Cruz V. M. V. Byrne P. F. Dierig D. A. (2013). Root traits contributing to plant productivity under drought. *Front. Plant Sci.* 4:442. 10.3389/fpls.2013.00442 24204374 PMC3817922

[B46] Cortijo S. Charoensawan V. Brestovitsky A. Buning R. Ravarani C. Rhodes D. (2017). Transcriptional regulation of the ambient temperature response by H2A.Z *nucleosomes and HSF*1 transcription factors in *Arabidopsis*. *Mol. Plant* 10 1258–1273. 10.1016/j.molp.2017.08.014 28893714 PMC6175055

[B47] Das A. Schneider H. Burridge J. Ascanio A. K. M. Wojciechowski T. Topp C. N. (2015). Digital imaging of root traits (DIRT): a high-throughput computing and collaboration platform for field-based root phenomics. *Plant Methods* 11:51. 10.1186/s13007-015-0093-3 26535051 PMC4630929

[B48] Dat J. F. Foyer C. H. Scott I. M. (1998). Changes in salicylic acid and antioxidants during induced thermotolerance in mustard seedlings. *Plant Physiol.* 118 1455–1461. 10.1104/pp.118.4.1455 9847121 PMC34763

[B49] de Dorlodot S. Forster B. Pagès L. Price A. Tuberosa R. Draye X. (2007). Root system architecture: opportunities and constraints for genetic improvement of crops. *Trends Plant Sci.* 12 474–481. 10.1016/j.tplants.2007.08.012 17822944

[B50] de Jong C. F. Takken F. L. W. Cai X. De Wit P. J. G. M. Joosten M. H. A. J. (2002). Attenuation of Cf-mediated defense responses at elevated temperatures correlates with a decrease in elicitor-binding sites. *Mol. Plant Microbe Interact.* 15 1040–1049. 10.1094/MPMI.2002.15.10.1040 12437302

[B51] Dello Ioio R. Nakamura K. Moubayidin L. Perilli S. Taniguchi M. Morita M. T. (2008). A genetic framework for the control of cell division and differentiation in the root meristem. *Science* 322 1380–1384. 10.1126/science.1164147 19039136

[B52] DeLucia E. H. Heckathorn S. A. Day T. A. (1992). Effects of soil temperature on growth, biomass allocation and resource acquisition of *Andropogon gerardii* Vitman. *New Phytol.* 120 543–549. 10.1111/j.1469-8137.1992.tb01804.x

[B53] Dempewolf H. Bordoni P. Rieseberg L. H. Engels J. M. M. (2010). Food security: crop species diversity. *Science* 328 169–170. 10.1126/science.328.5975.169-e 20378793

[B54] Dempewolf H. Eastwood R. J. Guarino L. Khoury C. K. Müller J. V. Toll J. (2014). Adapting agriculture to climate change: a global initiative to collect, conserve, and use crop wild relatives. *Agroecol. Sustain. Food Syst.* 38 369–377. 10.1080/21683565.2013.870629

[B55] Den Herder G. Van Isterdael G. Beeckman T. De Smet I. (2010). The roots of a new green revolution. *Trends Plant Sci.* 15 600–607. 10.1016/j.tplants.2010.08.009 20851036

[B56] Deryng D. Elliott J. Folberth C. Müller C. Pugh T. A. M. Boote K. J. (2016). Regional disparities in the beneficial effects of rising CO2 concentrations on crop water productivity. *Nat. Clim. Chang.* 6 786–790. 10.1038/nclimate2995

[B57] Dhaubhadel S. Browning K. S. Gallie D. R. Krishna P. (2002). Brassinosteroid functions to protect the translational machinery and heat-shock protein synthesis following thermal stress. *Plant J.* 29 681–691. 10.1046/j.1365-313X.2002.01257.x 12148527

[B58] Dhaubhadel S. Chaudhary S. Dobinson K. F. Krishna P. (1999). Treatment with 24-epibrassinolide, a brassinosteroid, increases the basic thermotolerance of *Brassica napus* and tomato seedlings. *Plant Mol. Biol.* 40 333–342. 10.1023/A:1006283015582 10412911

[B59] Dinneny J. R. (2019). Developmental responses to water and salinity in root systems. *Annu. Rev. Cell Dev. Biol.* 35 239–257. 10.1146/annurev-cellbio-100617-062949 31382759

[B60] Dong J. Gruda N. Lam S. K. Li X. Duan Z. (2018). Effects of elevated CO2 on nutritional quality of vegetables: a review. *Front. Plant Sci.* 9:924. 10.3389/fpls.2018.00924 30158939 PMC6104417

[B61] Driedonks N. Wolters-Arts M. Huber H. de Boer G. J. Vriezen W. Mariani C. (2018). Exploring the natural variation for reproductive thermotolerance in wild tomato species. *Euphytica* 214:67 10.1007/s10681-018-2150-2

[B62] Easterling D. R. Meehl G. A. Parmesan C. Changnon S. A. Karl T. R. Mearns L. O. (2000). Climate extremes: observations, modeling, and impacts. *Science* 289 2068–2074. 10.1126/science.289.5487.2068 11000103

[B63] El Hassouni K. Alahmad S. Belkadi B. Filali-Maltouf A. Hickey L. T. Bassi F. M. (2018). Root system architecture and its association with yield under different water regimes in durum wheat. *Crop Sci.* 58 2331–2346. 10.2135/cropsci2018.01.0076

[B64] Elad Y. Pertot I. (2014). Climate change impacts on plant pathogens and plant diseases. *J. Crop Improv.* 28 99–139. 10.1080/15427528.2014.865412

[B65] ElSayed A. I. Rafudeen M. S. Golldack D. (2014). Physiological aspects of raffinose family oligosaccharides in plants: protection against abiotic stress. *Plant Biol.* 16 1–8. 10.1111/plb.12053 23937337

[B66] Fahad S. Bajwa A. A. Nazir U. Anjum S. A. Farooq A. Zohaib A. (2017). Crop production under drought and heat stress: plant responses and management options. *Front. Plant Sci.* 8:1147. 10.3389/fpls.2017.01147 28706531 PMC5489704

[B67] FAO (2009a). *Food and Agriculture Organization of the United Nations (FAO), Global Agriculture Towards 2050.* Rome: FAO.

[B68] FAO (2009b). *Food and Agriculture Organization of the United Nations (FAO), How to Feed the World in 2050.* Rome: FAO.

[B69] Feng X. H. Zhang H. X. Ali M. Gai W. X. Cheng G. X. Yu Q. H. (2019). A small heat shock protein CaHsp25.9 positively regulates heat, salt, and drought stress tolerance in pepper (*Capsicum annuum* L.). *Plant Physiol. Biochem.* 142 151–162. 10.1016/j.plaphy.2019.07.001 31284139

[B70] Feng Z. De Marco A. Anav A. Gualtieri M. Sicard P. Tian H. (2019). Economic losses due to ozone impacts on human health, forest productivity and crop yield across China. *Environ. Int.* 131:e0104966. 10.1016/j.envint.2019.104966 31284106

[B71] Fernandez-Pozo N. Zheng Y. Snyder S. I. Nicolas P. Shinozaki Y. Fei Z. (2017). The tomato expression atlas. *Bioinformatics* 33 2397–2398. 10.1093/bioinformatics/btx190 28379331 PMC5860121

[B72] Foster G. Rahmstorf S. (2011). Global temperature evolution 1979–2010. *Environ. Res. Lett.* 6:044022 10.1088/1748-9326/6/4/044022

[B73] Friedli C. N. Abiven S. Fossati D. Hund A. (2019). Modern wheat semi-dwarfs root deep on demand: response of rooting depth to drought in a set of Swiss era wheats covering 100 years of breeding. *Euphytica* 215:85 10.1007/s10681-019-2404-7

[B74] Fuhrer J. (2003). Agroecosystem responses to combinations of elevated CO2, ozone, and global climate change. *Agric. Ecosyst. Environ.* 97 1–20. 10.1016/S0167-8809(03)00125-7

[B75] Fujita M. Fujita Y. Noutoshi Y. Takahashi F. Narusaka Y. Yamaguchi-Shinozaki K. (2006). Crosstalk between abiotic and biotic stress responses: a current view from the points of convergence in the stress signaling networks. *Curr. Opin. Plant Biol.* 9 436–442. 10.1016/j.pbi.2006.05.014 16759898

[B76] Füllner K. Temperton V. M. Rascher U. Jahnke S. Rist R. Schurr U. (2012). Vertical gradient in soil temperature stimulates development and increases biomass accumulation in barley. *Plant. Cell Environ.* 35 884–892. 10.1111/j.1365-3040.2011.02460.x 22070728

[B77] Garofalo P. Ventrella D. Kersebaum K. C. Gobin A. Trnka M. Giglio L. (2019). Water footprint of winter wheat under climate change: trends and uncertainties associated to the ensemble of crop models. *Sci. Total Environ.* 658 1186–1208. 10.1016/j.scitotenv.2018.12.279 30677982

[B78] Gill S. S. Tuteja N. (2010). Reactive oxygen species and antioxidant machinery in abiotic stress tolerance in crop plants. *Plant Physiol. Biochem.* 48 909–930. 10.1016/j.plaphy.2010.08.016 20870416

[B79] Giri A. Heckathorn S. Mishra S. Krause C. (2017). Heat stress decreases levels of nutrient-uptake and -assimilation proteins in tomato roots. *Plants* 6:6. 10.3390/plants6010006 28106834 PMC5371765

[B80] Godfray H. C. J. Beddington J. R. Crute I. R. Haddad L. Lawrence D. Muir J. F. (2010). Food security: the challenge of feeding 9 billion people. *Science* 327 812–818. 10.1126/science.1185383 20110467

[B81] Gong F. Wu X. Zhang H. Chen Y. Wang W. (2015). Making better maize plants for sustainable grain production in a changing climate. *Front. Plant Sci.* 6:835. 10.3389/fpls.2015.00835 26500671 PMC4593952

[B82] Gous P. W. Hickey L. Christopher J. T. Franckowiak J. Fox G. P. (2016). Discovery of QTL for stay-green and heat-stress in barley (*Hordeum vulgare*) grown under simulated abiotic stress conditions. *Euphytica* 207 305–317. 10.1007/s10681-015-1542-9

[B83] Guarino L. Lobell D. B. (2011). A walk on the wild side. *Nat. Clim. Chang.* 1 374–375. 10.1038/nclimate1272

[B84] Hanley M. E. Hartley F. C. Hayes L. Franco M. (2019). Simulated seawater flooding reduces oilseed rape growth, yield and progeny performance. *Ann. Bot.* 125 247–254. 10.1093/aob/mcz026 30918955 PMC7442384

[B85] Hasanuzzaman M. Nahar K. Bhuiyan T. F. Anee T. I. Inafuku M. Oku H. (2017). “Salicylic acid: an all-rounder in regulating abiotic stress responses in plants,” in *Phytohormones - Signaling Mechanisms and Crosstalk in Plant Development and Stress Responses*, ed. InTech (London: IntechOpen), 10.5772/intechopen.68213

[B86] Hecht V. L. Temperton V. M. Nagel K. A. Rascher U. Postma J. A. (2016). Sowing density: a neglected factor fundamentally affecting root distribution and biomass allocation of field grown spring barley (*Hordeum vulgare* L.). *Front. Plant Sci.* 7:944. 10.3389/fpls.2016.00944 27446171 PMC4923255

[B87] Heckathorn S. A. Giri A. Mishra S. Bista D. (2013). “Heat stress and roots,” in *Climate Change and Plant Abiotic Stress Tolerance*, eds Tuteja N. Gill S. S. (Weinheim: Wiley-VCH Verlag GmbH & Co), 109–136. 10.1002/9783527675265.ch05

[B88] Heckathorn S. A. Poeller G. J. Coleman J. S. Hallberg R. L. (1996). Nitrogen availability alters patterns of accumulation of heat stress-induced proteins in plants. *Oecologia* 105 413–418. 10.1007/BF00328745 28307115

[B89] Howell S. H. (2013). Endoplasmic reticulum stress responses in plants. *Annu. Rev. Plant Biol.* 64 477–499. 10.1146/annurev-arplant-050312-120053 23330794

[B90] Hu G. Li Z. Lu Y. Li C. Gong S. Yan S. (2017). Genome-wide association study identified multiple genetic loci on chilling resistance during germination in maize. *Sci. Rep.* 7:10840. 10.1038/s41598-017-11318-6 28883611 PMC5589824

[B91] Hua J. (2013). Modulation of plant immunity by light, circadian rhythm, and temperature. *Curr. Opin. Plant Biol.* 16 406–413. 10.1016/j.pbi.2013.06.017 23856082

[B92] Huang B. Xu Q. (2000). Root growth and nutrient element status of creeping bentgrass cultivars differing in heat tolerance as influenced by supraoptimal shoot and root temperatures. *J. Plant Nutr.* 23 979–990. 10.1080/01904160009382075

[B93] Huang B. R. Taylor H. M. McMichael B. L. (1991a). Growth and development of seminal and crown roots of wheat seedlings as affected by temperature. *Environ. Exp. Bot.* 31 471–477.

[B94] Huang B. R. Taylor H. M. McMichael B. L. (1991b). Effects of temperature on the development of metaxylem in primary wheat roots and its hydraulic consequence. *Ann. Bot.* 67 163–166. 10.1093/oxfordjournals.aob.a088115

[B95] Hund A. Fracheboud Y. Soldati A. Stamp P. (2008). Cold tolerance of maize seedlings as determined by root morphology and photosynthetic traits. *Eur. J. Agron.* 28 178–185. 10.1016/j.eja.2007.07.003

[B96] Huot B. Castroverde C. D. M. Velásquez A. C. Hubbard E. Pulman J. A. Yao J. (2017). Dual impact of elevated temperature on plant defence and bacterial virulence in *Arabidopsis*. *Nat. Commun.* 8:1808. 10.1038/s41467-017-01674-2 29180698 PMC5704021

[B97] Hussain H. A. Men S. Hussain S. Chen Y. Ali S. Zhang S. (2019). Interactive effects of drought and heat stresses on morpho-physiological attributes, yield, nutrient uptake and oxidative status in maize hybrids. *Sci. Rep.* 9:7. 10.1038/s41598-019-40362-7 30846745 PMC6405865

[B98] Iglesias-Acosta M. Martínez-Ballesta M. C. Teruel J. A. Carvajal M. (2010). The response of broccoli plants to high temperature and possible role of root aquaporins. *Environ. Exp. Bot.* 68 83–90. 10.1016/j.envexpbot.2009.10.007

[B99] Iizumi T. Ramankutty N. (2015). How do weather and climate influence cropping area and intensity? *Glob. Food Sec.* 4 46–50. 10.1016/j.gfs.2014.11.003

[B100] Ionenko I. F. Anisimov A. V. Dautova N. R. (2010). Effect of temperature on water transport through aquaporins. *Biol. Plant.* 54 488–494. 10.1007/s10535-010-0086-z

[B101] IPCC (2014). “Climate change 2014: synthesis report,” in *Contribution of Working Groups I, II and III to the Fifth Assessment Report of the Intergovernmental Panel on Climate Change*, eds Pachauri R. K. Meyer L. A. (Geneva: IPCC).

[B102] Isayenkov S. V. Maathuis F. J. M. (2019). Plant salinity stress: many unanswered questions remain. *Front. Plant Sci.* 10:80. 10.3389/fpls.2019.00080 30828339 PMC6384275

[B103] Jagadish S. V. K. Murty M. V. R. Quick W. P. (2015). Rice responses to rising temperatures - challenges, perspectives and future directions. *Plant. Cell Environ.* 38 1686–1698. 10.1111/pce.12430 25142172

[B104] Jagadish S. V. K. Muthurajan R. Oane R. Wheeler T. R. Heuer S. Bennett J. (2010). Physiological and proteomic approaches to address heat tolerance during anthesis in rice (Oryza sativa L.). *J. Exp. Bot.* 61 143–156. 10.1093/jxb/erp289 19858118 PMC2791117

[B105] Jamil M. Ali A. Gul A. Ghafoor A. Napar A. A. Ibrahim A. M. H. (2019). Genome-wide association studies of seven agronomic traits under two sowing conditions in bread wheat. *BMC Plant Biol.* 19:149. 10.1186/s12870-019-1754-6 31003597 PMC6475106

[B106] Jia J. Zhou J. Shi W. Cao X. Luo J. Polle A. (2017). Comparative transcriptomic analysis reveals the roles of overlapping heat-/drought-responsive genes in poplars exposed to high temperature and drought. *Sci. Rep.* 7:43215. 10.1038/srep43215 28233854 PMC5324098

[B107] Jia Z. Liu Y. Gruber B. D. Neumann K. Kilian B. Graner A. (2019). Genetic dissection of root system architectural traits in spring barley. *Front. Plant Sci.* 10:400. 10.3389/fpls.2019.00400 31001309 PMC6454135

[B108] Joshi M. Fogelman E. Belausov E. Ginzberg I. (2016). Potato root system development and factors that determine its architecture. *J. Plant Physiol.* 205 113–123. 10.1016/j.jplph.2016.08.014 27669493

[B109] Ju C. Zhang W. Liu Y. Gao Y. Wang X. Yan J. (2018). Genetic analysis of seedling root traits reveals the association of root trait with other agronomic traits in maize. *BMC Plant Biol* 18:171. 10.1186/s12870-018-1383-5 30111287 PMC6094888

[B110] Jung T. Burgess T. I. (2009). Re-evaluation of *Phytophthora citricola* isolates from multiple woody hosts in Europe and North America reveals a new species, *Phytophthora plurivora* sp. nov. *Persoonia Mol. Phylogeny Evol. Fungi* 22 95–110. 10.3767/003158509X442612 20198142 PMC2789536

[B111] Kang Z. Qin T. Zhao Z. (2019). Overexpression of the zinc finger protein gene OsZFP350 improves root development by increasing resistance to abiotic stress in rice. *Acta Biochim. Pol.* 66 183–190. 10.18388/abp.2018_2765 31125390

[B112] Karanja B. K. Xu L. Wang Y. Muleke E. M. Jabir B. M. Xie Y. (2017). Genome-wide characterization and expression profiling of NAC transcription factor genes under abiotic stresses in radish (*Raphanus sativus* L.). *PeerJ* 5:e4172. 10.7717/peerj.4172 29259849 PMC5733918

[B113] Keleş Y. Öncel I. (2002). Response of antioxidative defence system to temperature and water stress combinations in wheat seedlings. *Plant Sci.* 163 783–790. 10.1016/S0168-9452(02)00213-3

[B114] Keyes S. D. Daly K. R. Gostling N. J. Jones D. L. Talboys P. Pinzer B. R. (2013). High resolution synchrotron imaging of wheat root hairs growing in soil and image based modelling of phosphate uptake. *New Phytol.* 198 1023–1029. 10.1111/nph.12294 23600607

[B115] Khan M. I. R. Fatma M. Per T. S. Anjum N. A. Khan N. A. (2015). Salicylic acid-induced abiotic stress tolerance and underlying mechanisms in plants. *Front. Plant Sci.* 6:462. 10.3389/fpls.2015.00462 26175738 PMC4485163

[B116] Khatun K. Robin A. H. K. Park J.-I. Nath U. K. Kim C. K. Lim K.-B. (2017). Molecular characterization and expression profiling of tomato GRF transcription factor family genes in response to abiotic stresses and phytohormones. *Int. J. Mol. Sci.* 18:1056. 10.3390/ijms18051056 28505092 PMC5454968

[B117] Khoury C. K. Castañeda-Alvarez N. P. Achicanoy H. A. Sosa C. C. Bernau V. Kassa M. T. (2015). Crop wild relatives of pigeonpea [*Cajanus cajan* (L.) Millsp.]: distributions, ex situ conservation status, and potential genetic resources for abiotic stress tolerance. *Biol. Conserv.* 184 259–270. 10.1016/j.biocon.2015.01.032

[B118] Kieber J. J. Schaller G. E. (2018). Cytokinin signaling in plant development. *Development* 145 344. 10.1242/dev.149344 29487105

[B119] Kilasi N. L. Singh J. Vallejos C. E. Ye C. Jagadish S. V. K. Kusolwa P. (2018). Heat Stress Tolerance in rice (*Oryza sativa* L.): Identification of quantitative trait loci and candidate genes for seedling growth under heat stress. *Front. Plant Sci.* 9:1578. 10.3389/fpls.2018.01578 30443261 PMC6221968

[B120] Killi D. Bussotti F. Raschi A. Haworth M. (2017). Adaptation to high temperature mitigates the impact of water deficit during combined heat and drought stress in C3 sunflower and C4 maize varieties with contrasting drought tolerance. *Physiol. Plant.* 159 130–147. 10.1111/ppl.12490 27535211

[B121] Kim J. M. Sasaki T. Ueda M. Sako K. Seki M. (2015). Chromatin changes in response to drought, salinity, heat, and cold stresses in plants. *Front. Plant Sci.* 6:114. 10.3389/fpls.2015.00114 25784920 PMC4345800

[B122] Koevoets I. T. Venema J. H. Elzenga J. T. M. Testerink C. (2016). Roots withstanding their environment: exploiting root system architecture responses to abiotic stress to improve crop tolerance. *Front. Plant Sci.* 7:1335. 10.3389/fpls.2016.01335 27630659 PMC5005332

[B123] Koh S. Lee S. C. Kim M. K. Koh J. H. Lee S. An G. (2007). T-DNA tagged knockout mutation of rice OsGSK1, an orthologue of *Arabidopsis* BIN2, with enhanced tolerance to various abiotic stresses. *Plant Mol. Biol.* 65 453–466. 10.1007/s11103-007-9213-4 17690841

[B124] Kosová K. Vítámvás P. Urban M. O. Prášil I. T. Renaut J. (2018). Plant abiotic stress proteomics: the major factors determining alterations in cellular proteome. *Front. Plant Sci.* 9:122. 10.3389/fpls.2018.00122 29472941 PMC5810178

[B125] Kruszka K. Pacak A. Swida-Barteczka A. Nuc P. Alaba S. Wroblewska Z. (2014). Transcriptionally and post-transcriptionally regulated microRNAs in heat stress response in barley. *J. Exp. Bot.* 65 6123–6135. 10.1093/jxb/eru353 25183744 PMC4203144

[B126] Kumar S. V. Wigge P. A. (2010). H2A.Z-Containing nucleosomes mediate the thermosensory response in *Arabidopsis*. *Cell* 140 136–147. 10.1016/j.cell.2009.11.006 20079334

[B127] Kwasniewski M. Daszkowska-Golec A. Janiak A. Chwialkowska K. Nowakowska U. Sablok G. (2016). Transcriptome analysis reveals the role of the root hairs as environmental sensors to maintain plant functions under water-deficiency conditions. *J. Exp. Bot.* 67 1079–1094. 10.1093/jxb/erv498 26585228 PMC4753848

[B128] Laloum T. Martín G. Duque P. (2018). Alternative splicing control of abiotic stress responses. *Trends Plant Sci.* 23 140–150. 10.1016/j.tplants.2017.09.019 29074233

[B129] Lämke J. Bäurle I. (2017). Epigenetic and chromatin-based mechanisms in environmental stress adaptation and stress memory in plants. *Genome Biol.* 18:124. 10.1186/s13059-017-1263-6 28655328 PMC5488299

[B130] Larkindale J. Hall J. D. Knight M. R. Vierling E. (2005). Heat stress phenotypes of *Arabidopsis mutants* implicate multiple signaling pathways in the acquisition of thermotolerance. *Plant Physiol.* 138 882–897. 10.1104/pp.105.062257 15923322 PMC1150405

[B131] Larkindale J. Huang B. (2004). Thermotolerance and antioxidant systems in *Agrostis stolonifera*: involvement of salicylic acid, abscisic acid, calcium, hydrogen peroxide, and ethylene. *J. Plant Physiol.* 161 405–413. 10.1078/0176-1617-01239 15128028

[B132] Lean J. L. Rind D. H. (2009). How will Earth’s surface temperature change in future decades? *Geophys. Res. Lett.* 36:e038932 10.1029/2009GL038932

[B133] Lesk C. Rowhani P. Ramankutty N. (2016). Influence of extreme weather disasters on global crop production. *Nature* 529 84–87. 10.1038/nature16467 26738594

[B134] Li W. Dong J. Cao M. Gao X. Wang D. Liu B. (2019). Genome-wide identification and characterization of HD-ZIP genes in potato. *Gene* 697 103–117. 10.1016/j.gene.2019.02.024 30776460

[B135] Li X. Chen R. Chu Y. Huang J. Jin L. Wang G. (2018). Overexpression of RCc3 improves root system architecture and enhances salt tolerance in rice. *Plant Physiol. Biochem.* 130 566–576. 10.1016/j.plaphy.2018.08.008 30103148

[B136] Li X. Guo Z. Lv Y. Cen X. Ding X. Wu H. (2017). Genetic control of the root system in rice under normal and drought stress conditions by genome-wide association study. *PLoS Genet.* 13:889. 10.1371/journal.pgen.1006889 28686596 PMC5521850

[B137] Li Y. Wang G. Xu Z. Li J. Sun M. Guo J. (2017). Organization and regulation of soybean SUMOylation system under abiotic stress conditions. *Front. Plant Sci.* 8:1458. 10.3389/fpls.2017.01458 28878795 PMC5573446

[B138] Li Z.-G. Yang S.-Z. Long W.-B. Yang G.-X. Shen Z.-Z. (2013). Hydrogen sulphide may be a novel downstream signal molecule in nitric oxide-induced heat tolerance of maize (*Zea mays* L.) seedlings. *Plant. Cell Environ.* 36 1564–1572. 10.1111/pce.12092 23489239

[B139] Lin M. Y. Chai K. H. Ko S. S. Kuang L. Y. Lur H. S. Charng Y. Y. (2014). A positive feedback loop between HEAT SHOCK PROTEIN101 and HEAT STRESS-ASSOCIATED 32-KD PROTEIN modulates long-term acquired thermotolerance illustrating diverse heat stress responses in rice varieties. *Plant Physiol.* 164 2045–2053. 10.1104/pp.113.229609 24520156 PMC3982761

[B140] Lin Z. Zhong S. Grierson D. (2009). Recent advances in ethylene research. *J. Exp. Bot.* 60 3311–3336. 10.1093/jxb/erp204 19567479

[B141] Liu B. Asseng S. Müller C. Ewert F. Elliott J. Lobell D. B. (2016). Similar estimates of temperature impacts on global wheat yield by three independent methods. *Nat. Clim. Chang.* 6 1130–1136. 10.1038/nclimate3115

[B142] Liu B. Liu L. Tian L. Cao W. Zhu Y. Asseng S. (2014). Post-heading heat stress and yield impact in winter wheat of China. *Glob. Chang. Biol.* 20 372–381. 10.1111/gcb.12442 24259291

[B143] Liu B. Martre P. Ewert F. Porter J. R. Challinor A. J. Müller C. (2019). Global wheat production with 1.5 and 2.0°C above pre-industrial warming. *Glob. Chang. Biol.* 25 1428–1444. 10.1111/gcb.14542 30536680

[B144] Liu H.-T. Liu Y.-P. Huang W.-D. (2008). Root-fed salicylic acid in grape involves the response caused by aboveground high temperature. *J. Integr. Plant Biol.* 50 761–767. 10.1111/j.1744-7909.2008.00640.x 18713417

[B145] Liu J. Feng L. Li J. He Z. (2015). Genetic and epigenetic control of plant heat responses. *Front. Plant Sci.* 6:267. 10.3389/fpls.2015.00267 25964789 PMC4408840

[B146] Liu J. Howell S. H. (2016). Managing the protein folding demands in the endoplasmic reticulum of plants. *New Phytol.* 211 418–428. 10.1111/nph.13915 26990454

[B147] Liu Q. Yan S. Yang T. Zhang S. Chen Y.-Q. Liu B. (2017). Small RNAs in regulating temperature stress response in plants. *J. Integr. Plant Biol.* 59 774–791. 10.1111/jipb.12571 28731217

[B148] Lobell D. B. Burke M. B. (2008). Why are agricultural impacts of climate change so uncertain? the importance of temperature relative to precipitation. *Environ. Res. Lett.* 3:7 10.1088/1748-9326/3/3/034007

[B149] Lobell D. B. Field C. B. (2007). Global scale climate-crop yield relationships and the impacts of recent warming. *Environ. Res. Lett.* 2:14002 10.1088/1748-9326/2/1/014002

[B150] Lobell D. B. Schlenker W. Costa-Roberts J. (2011). Climate trends and global crop production since 1980. *Science* 333 616–620. 10.1126/science.1204531 21551030

[B151] Long S. P. Ainsworth E. A. Rogers A. Ort D. R. (2004). Rising atmospheric carbon dioxide: plants FACE the future. *Annu. Rev. Plant Biol.* 55 591–628. 10.1146/annurev.arplant.55.031903.141610 15377233

[B152] Long S. P. Ort D. R. (2010). More than taking the heat: crops and global change. *Curr. Opin. Plant Biol.* 13 240–247. 10.1016/j.pbi.2010.04.008 20494611

[B153] Lynch J. (1995). Root architecture and plant productivity. *Plant Physiol.* 109 7–13. 10.1104/pp.109.1.7 12228579 PMC157559

[B154] Lynch J. P. (2013). Steep, cheap and deep: an ideotype to optimize water and N acquisition by maize root systems. *Ann. Bot.* 112 347–357. 10.1093/aob/mcs293 23328767 PMC3698384

[B155] Ma L. Shi Y. Siemianowski O. Yuan B. Egner T. K. Mirnezami S. V. (2019). Hydrogel-based transparent soils for root phenotyping in vivo. *Proc. Natl. Acad. Sci. U. S. A.* 166 11063–11068. 10.1073/pnas.1820334116 31088969 PMC6561166

[B156] Ma P. Chen X. Liu C. Xia Z. Song Y. Zeng C. (2018). MePHD1 as a PHD-Finger protein negatively regulates adp-glucose pyrophosphorylase small subunit1a gene in cassava. *Int. J. Mol. Sci.* 19:2831. 10.3390/ijms19092831 30235813 PMC6164933

[B157] Maccaferri M. El-Feki W. Nazemi G. Salvi S. Canè M. A. Colalongo M. C. (2016). Prioritizing quantitative trait loci for root system architecture in tetraploid wheat. *J. Exp. Bot.* 67 1161–1178. 10.1093/jxb/erw039 26880749 PMC4753857

[B158] Macduff J. H. Wild A. Hopper M. J. Dhanoa M. S. (1986). Effects of temperature on parameters of root growth relevant to nutrient uptake: measurements on oilseed rape and barley grown in flowing nutrient solution. *Plant Soil* 94 321–332. 10.1007/BF02374326

[B159] Macková H. Hronková M. Dobrá J. Tureèková V. Novák O. Lubovská Z. (2013). Enhanced drought and heat stress tolerance of tobacco plants with ectopically enhanced cytokinin oxidase/dehydrogenase gene expression. *J. Exp. Bot.* 64 2805–2815. 10.1093/jxb/ert131 23669573 PMC3741687

[B160] Mahalingam R. (2015). “Consideration of combined stress: a crucial paradigm for improving multiple stress tolerance in plants,” in *Combined Stresses in Plants: Physiological, Molecular, and Biochemical Aspects*, ed. Mahalingam R. (Cham: Springer International Publishing), 1–26. 10.1007/978-3-319-07899-1_1

[B161] Mahalingam R. Bregitzer P. (2019). Impact on physiology and malting quality of barley exposed to heat, drought and their combination during different growth stages under controlled environment. *Physiol. Plant.* 165 277–289. 10.1111/ppl.12841 30238998

[B162] Mahmud K. P. Holzapfel B. P. Guisard Y. Smith J. P. Nielsen S. Rogiers S. Y. (2018). Circadian regulation of grapevine root and shoot growth and their modulation by photoperiod and temperature. *J. Plant Physiol.* 222 86–93. 10.1016/j.jplph.2018.01.006 29407553

[B163] Martins S. Montiel-Jorda A. Cayrel A. Huguet S. Roux C. P. Le Ljung K. (2017). Brassinosteroid signaling-dependent root responses to prolonged elevated ambient temperature. *Nat. Commun.* 8:309. 10.1038/s41467-017-00355-4 28827608 PMC5567177

[B164] Maulana F. Ayalew H. Anderson J. D. Kumssa T. T. Huang W. Ma X.-F. (2018). Genome-wide association mapping of seedling heat tolerance in winter wheat. *Front. Plant Sci.* 9:1272. 10.3389/fpls.2018.01272 30233617 PMC6131858

[B165] Maurel C. Boursiac Y. Luu D. T. Santoni V. Shahzad Z. Verdoucq L. (2015). Aquaporins in plants. *Physiol. Rev.* 95 1321–1358. 10.1152/physrev.00008.2015 26336033

[B166] McClung C. R. Davis S. J. (2010). Ambient thermometers in plants: From physiological outputs towards mechanisms of thermal sensing. *Curr. Biol.* 20 R1086–R1092. 10.1016/j.cub.2010.10.035 21172632

[B167] Meister R. Rajani M. S. Ruzicka D. Schachtman D. P. (2014). Challenges of modifying root traits in crops for agriculture. *Trends Plant Sci.* 19 779–788. 10.1016/j.tplants.2014.08.005 25239776

[B168] Mendes R. Kruijt M. De Bruijn I. Dekkers E. Van Der Voort M. Schneider J. H. M. (2011). Deciphering the rhizosphere microbiome for disease-suppressive bacteria. *Science* 332 1097–1100. 10.1126/science.1203980 21551032

[B169] Mills G. Hayes F. Jones M. L. Cinderby S. (2007). Identifying ozone-sensitive communities of (semi-) natural vegetation suitable for mapping exceedance of critical levels. *Environ. Pollut.* 146 736–743. 10.1016/j.envpol.2006.04.005 16781803

[B170] Mittler R. Finka A. Goloubinoff P. (2012). How do plants feel the heat? *Trends Biochem. Sci.* 37 118–125. 10.1016/j.tibs.2011.11.007 22236506

[B171] Monirul Qader Mirza M. (2002). Global warming and changes in the probability of occurrence of floods in Bangladesh and implications. *Glob. Environ. Chang.* 12 127–138. 10.1016/S0959-3780(02)00002-X

[B172] Morales D. Rodríguez P. Dell’Amico J. Nicolás E. Torrecillas A. Sánchez-Blanco M. J. (2003). High-temperature preconditioning and thermal shock imposition affects water relations, gas exchange and root hydraulic conductivity in tomato. *Biol. Plant.* 47 203–208. 10.1023/B:BIOP.0000022252.70836.fc

[B173] Morita S. Wada H. Matsue Y. (2016). Countermeasures for heat damage in rice grain quality under climate change. *Plant Prod. Sci.* 19 1–11. 10.1080/1343943X.2015.1128114

[B174] Morrison M. J. Stewart D. W. (2002). Heat stress during flowering in summer Brassica. *Crop Sci.* 42 797–803. 10.2135/cropsci2002.7970

[B175] Müller M. Munné-Bosch S. (2015). Ethylene response factors: a key regulatory hub in hormone and stress signaling. *Plant Physiol.* 169 32–41. 10.1104/pp.15.00677 26103991 PMC4577411

[B176] Nagel K. A. Kastenholz B. Jahnke S. van Dusschoten D. Aach T. Mühlich M. (2009). Temperature responses of roots: impact on growth, root system architecture and implications for phenotyping. *Funct. Plant Biol.* 36 947 10.1071/FP09184 32688706

[B177] Nazar R. Iqbal N. Umar S. (2017). “Heat stress tolerance in plants: action of salicylic acid,” in *Salicylic Acid: A Multifaceted Hormone*, eds Nazar R. Iqbal N. Khan N. (Singapore: Springer), 145–161. 10.1007/978-981-10-6068-7_8

[B178] Neill E. M. Byrd M. C. R. Billman T. Brandizzi F. Stapleton A. E. (2019). Plant growth regulators interact with elevated temperature to alter heat stress signaling via the Unfolded Protein Response in maize. *Sci. Rep.* 9:839. 10.1038/s41598-019-46839-9 31316112 PMC6637120

[B179] Nieto-Sotelo J. Ho T.-H. D. (1986). Effect of heat shock on the metabolism of glutathione in maize roots. *Plant Physiol.* 82 1031–1035. 10.1104/pp.82.4.1031 16665130 PMC1056253

[B180] Nieto-Sotelo J. Martínez L. M. Ponce G. Cassab G. I. Alagón A. Meeley R. B. (2002). Maize HSP101 plays important roles in both induced and basal thermotolerance and primary root growth. *Plant Cell* 14 1621–1633. 10.1105/tpc.010487 12119379 PMC150711

[B181] Nolan T. Chen J. Yin Y. (2017). Cross-talk of brassinosteroid signaling in controlling growth and stress responses. *Biochem. J.* 474 2641–2661. 10.1042/BCJ20160633 28751549 PMC6296487

[B182] Nolan T. Vukasinovic N. Liu D. Russinova E. Yin Y. (2019). Brassinosteroids: multi-dimensional regulators of plant growth, development, and stress responses. *Plant Cell* 32 295–318. 10.1105/tpc.19.00335 31776234 PMC7008487

[B183] Ohama N. Sato H. Shinozaki K. Yamaguchi-Shinozaki K. (2017). Transcriptional regulatory network of plant heat stress response. *Trends Plant Sci.* 22 53–65. 10.1016/j.tplants.2016.08.015 27666516

[B184] Oladzad A. Porch T. Rosas J. C. Moghaddam S. M. Beaver J. Beebe S. E. (2019). Single and multi-trait GWAS identify genetic factors associated with production traits in common bean under abiotic stress environments. *G3 Genes Genomes Genet.* 9 1881–1892. 10.1534/g3.119.400072 31167806 PMC6553540

[B185] Orosa-Puente B. Leftley N. von Wangenheim D. Banda J. Srivastava A. K. Hill K. (2018). Root branching toward water involves posttranslational modification of transcription factor ARF7. *Science* 362 1407–1410. 10.1126/science.aau3956 30573626

[B186] Pardales J. R. Banoc D. M. Yamauchi A. Iijima M. Kono Y. (1999). Root system development of cassava and sweetpotato during early growth stage as affected by high root zone temperature. *Plant Prod. Sci.* 2 247–251. 10.1626/pps.2.247

[B187] Pardales J. R. Kono Y. Yamauchi A. (1992). Epidermal cell elongation in sorghum seminal roots exposed to high root-zone temperature. *Plant Sci.* 81 143–146. 10.1016/0168-9452(92)90035-K

[B188] Parts K. Tedersoo L. Schindlbacher A. Sigurdsson B. D. Leblans N. I. W. Oddsdóttir E. S. (2019). Acclimation of fine root systems to soil warming: comparison of an experimental setup and a natural soil temperature gradient. *Ecosystems* 22 457–472. 10.1007/s10021-018-0280-y

[B189] Penfield S. (2008). Temperature perception and signal transduction in plants. *New Phytol.* 179 615–628. 10.1111/j.1469-8137.2008.02478.x 18466219

[B190] Pfeifer J. Faget M. Walter A. Blossfeld S. Fiorani F. Schurr U. (2014). Spring barley shows dynamic compensatory root and shoot growth responses when exposed to localised soil compaction and fertilisation. *Funct. Plant Biol.* 41 581–597. 10.1071/FP13224 32481015

[B191] Philippot L. Raaijmakers J. M. Lemanceau P. Van Der Putten W. H. (2013). Going back to the roots: the microbial ecology of the rhizosphere. *Nat. Rev. Microbiol.* 11 789–799. 10.1038/nrmicro3109 24056930

[B192] Pinto R. S. Reynolds M. P. (2015). Common genetic basis for canopy temperature depression under heat and drought stress associated with optimized root distribution in bread wheat. *Theor. Appl. Genet.* 128 575–585. 10.1007/s00122-015-2453-9 25707766 PMC4361760

[B193] Pinto R. S. Reynolds M. P. Mathews K. L. McIntyre C. L. Olivares-Villegas J. J. Chapman S. C. (2010). Heat and drought adaptive QTL in a wheat population designed to minimize confounding agronomic effects. *Theor. Appl. Genet.* 121 1001–1021. 10.1007/s00122-010-1351-4 20523964 PMC2938441

[B194] Plieth C. Hansen U.-P. Knight H. Knight M. R. (1999). Temperature sensing by plants: the primary characteristics of signal perception and calcium response. *Plant J.* 18 491–497. 10.1046/j.1365-313X.1999.00471.x 10417699

[B195] Prasad P. V. V. Boote K. J. Allen L. H. Sheehy J. E. Thomas J. M. G. (2006). Species, ecotype and cultivar differences in spikelet fertility and harvest index of rice in response to high temperature stress. *F. Crop. Res.* 95 398–411. 10.1016/j.fcr.2005.04.008

[B196] Prasad P. V. V. Staggenborg S. A. Ristic Z. Ahuja L. R. Reddy V. R. Saseendran S. A. (2008). *Impacts of Drought And/Or Heat Stress On Physiological, Developmental, Growth, And Yield Processes Of Crop Plants.* Madison, WI: American Society of Agronomy, 10.2134/advagricsystmodel1.c11

[B197] Pregitzer K. S. Zak D. R. Maziasz J. DeForest J. Curtis P. S. Lussenhop J. (2000). Interactive effects of atmospheric CO2 and soil-N availability on fine roots of Populus tremuloides. *Ecol. Appl.* 10 18–33.

[B198] Ps S. Sv A. M. Prakash C. Mk R. Tiwari R. Mohapatra T. (2017). High resolution mapping of QTLs for heat tolerance in rice using a 5K SNP Array. *Rice* 10:167. 10.1186/s12284-017-0167-0 28584974 PMC5459777

[B199] Qaseem M. F. Qureshi R. Shaheen H. (2019). Effects of pre-anthesis drought, heat and their combination on the growth, yield and physiology of diverse wheat (*Triticum aestivum* L.) genotypes varying in sensitivity to heat and drought stress. *Sci. Rep.* 9:477. 10.1038/s41598-019-43477-z 31061444 PMC6502848

[B200] Qiao S. Fang Y. Wu A. Xu B. Zhang S. Deng X. (2019). Dissecting root trait variability in maize genotypes using the semi-hydroponic phenotyping platform. *Plant Soil* 439 75–90. 10.1007/s11104-018-3803-6

[B201] Qin L. He J. Lee S. K. Dodd I. C. (2007). An assessment of the role of ethylene in mediating lettuce (*Lactuca sativa*) root growth at high temperatures. *J. Exp. Bot.* 58 3017–3024. 10.1093/jxb/erm156 17728295

[B202] Queitsch C. Hong S. W. Vierling E. Lindquist S. (2000). Heat shock protein 101 plays a crucial role in thermotolerance in *Arabidopsis*. *Plant Cell* 12 479–492. 10.1105/tpc.12.4.479 10760238 PMC139847

[B203] Quint M. Delker C. Franklin K. A. Wigge P. A. Halliday K. J. van Zanten M. (2016). Molecular and genetic control of plant thermomorphogenesis. *Nat. Plants* 2:15190. 10.1038/nplants.2015.190 27250752

[B204] Ramírez-González R. H. Borrill P. Lang D. Harrington S. A. Brinton J. Venturini L. (2018). The transcriptional landscape of polyploid wheat. *Science* 361:6403. 10.1126/science.aar6089 30115782

[B205] Rasul I. Nadeem H. Siddique M. H. Atif R. M. Ali M. A. Umer A. (2017). Plants sensory-response mechanisms for salinity and heat stress. *J. Anim. Plant Sci.* 27 490–502.

[B206] Ray D. K. Mueller N. D. West P. C. Foley J. A. (2013). Yield trends are insufficient to double global crop production by 2050. *PLoS One* 8:e66428. 10.1371/journal.pone.0066428 23840465 PMC3686737

[B207] Ray D. K. West P. C. Clark M. Gerber J. S. Prishchepov A. V. Chatterjee S. (2019). Climate change has likely already affected global food production. *PLoS One* 14:e0217148. 10.1371/journal.pone.0217148 31150427 PMC6544233

[B208] Reimer R. Stich B. Melchinger A. E. Schrag T. A. Sørensen A. P. Stamp P. (2013). Root response to temperature extremes: association mapping of temperate maize (*Zea mays* L). *Maydica* 58 156–168.

[B209] Ribeiro P. R. Fernandez L. G. de Castro R. D. Ligterink W. Hilhorst H. W. (2014). Physiological and biochemical responses of *Ricinus communis* seedlings to different temperatures: a metabolomics approach. *BMC Plant Biol.* 14:223. 10.1186/s12870-014-0223-5 25109402 PMC4236761

[B210] Ristova D. Busch W. (2014). Natural variation of root traits: from development to nutrient uptake. *Plant Physiol.* 166 518–527. 10.1104/pp.114.244749 25104725 PMC4213084

[B211] Ristova D. Giovannetti M. Metesch K. Busch W. (2018). Natural genetic variation shapes root system responses to phytohormones in *Arabidopsis*. *Plant J.* 96 468–481. 10.1111/tpj.14034 30030851 PMC6220887

[B212] Rivas-San Vicente M. Plasencia J. (2011). Salicylic acid beyond defence: Its role in plant growth and development. *J. Exp. Bot.* 62 3321–3338. 10.1093/jxb/err031 21357767

[B213] Rivero R. M. Mestre T. C. Mittler R. Rubio F. Garcia-sanchez F. Martinez V. (2014). The combined effect of salinity and heat reveals a specific physiological, biochemical and molecular response in tomato plants. *Plant. Cell Environ.* 37 1059–1073. 10.1111/pce.12199 24028172

[B214] Robin A. H. K. Matthew C. Uddin M. J. Bayazid K. N. (2016). Salinity-induced reduction in root surface area and changes in major root and shoot traits at the phytomer level in wheat. *J. Exp. Bot.* 67 3719–3729. 10.1093/jxb/erw064 26951370

[B215] Robinson H. Kelly A. Fox G. Franckowiak J. Borrell A. Hickey L. (2018). Root architectural traits and yield: exploring the relationship in barley breeding trials. *Euphytica* 214:151 10.1007/s10681-018-2219-y

[B216] Rolli E. Marasco R. Vigani G. Ettoumi B. Mapelli F. Deangelis M. L. (2015). Improved plant resistance to drought is promoted by the root-associated microbiome as a water stress-dependent trait. *Environ. Microbiol.* 17 316–331. 10.1111/1462-2920.12439 24571749

[B217] Saadi S. Todorovic M. Tanasijevic L. Pereira L. S. Pizzigalli C. Lionello P. (2015). Climate change and Mediterranean agriculture: Impacts on winter wheat and tomato crop evapotranspiration, irrigation requirements and yield. *Agric. Water Manag.* 147 103–115. 10.1016/j.agwat.2014.05.008

[B218] Sahni S. Prasad B. D. Liu Q. Grbic V. Sharpe A. Singh S. P. (2016). Overexpression of the brassinosteroid biosynthetic gene DWF4 in Brassica napus simultaneously increases seed yield and stress tolerance. *Sci. Rep.* 6:28298. 10.1038/srep28298 27324083 PMC4915011

[B219] Salvi P. Kamble N. U. Majee M. (2018). Stress-inducible galactinol synthase of chickpea (CaGolS) is implicated in heat and oxidative stress tolerance through reducing stress-induced excessive reactive oxygen species accumulation. *Plant Cell Physiol.* 59 155–166. 10.1093/pcp/pcx170 29121266

[B220] Sani S. G. A. S. Chang P. L. Zubair A. Carrasquilla-Garcia N. Cordeiro M. Penmetsa R. V. (2018). Genetic diversity, population structure, and genetic correlation with climatic variation in chickpea (*Cicer arietinum*) landraces from Pakistan. *Plant Genome* 11:67. 10.3835/plantgenome2017.08.0067 29505627 PMC12810059

[B221] Santhanam R. Luu V. T. Weinhold A. Goldberg J. Oh Y. Baldwin I. T. (2015). Native root-associated bacteria rescue a plant from a sudden-wilt disease that emerged during continuous cropping. *Proc. Natl. Acad. Sci. U.S.A.* 112 E5013–E5120. 10.1073/pnas.1505765112 26305938 PMC4568709

[B222] Saraswat S. Yadav A. K. Sirohi P. Singh N. K. (2017). Role of epigenetics in crop improvement: water and heat stress. *J. Plant Biol.* 60 231–240.

[B223] Sattelmacher B. Marschner H. Kühne R. (1990). Effects of the temperature of the rooting zone on the growth and development of roots of potato (*Solanum tuberosum*). *Ann. Bot.* 65 27–36. 10.2307/42771369

[B224] Sekmen A. H. Ozgur R. Uzilday B. Turkan I. (2014). Reactive oxygen species scavenging capacities of cotton (*Gossypium hirsutum*) cultivars under combined drought and heat induced oxidative stress. *Environ. Exp. Bot.* 99 141–149. 10.1016/j.envexpbot.2013.11.010

[B225] Selote D. S. Khanna-Chopra R. (2010). Antioxidant response of wheat roots to drought acclimation. *Protoplasma* 245 153–163. 10.1007/s00709-010-0169-x 20559854

[B226] Senapati N. Brown H. E. Semenov M. A. (2019). Raising genetic yield potential in high productive countries: designing wheat ideotypes under climate change. *Agric. For. Meteorol.* 271 33–45. 10.1016/j.agrformet.2019.02.025 31217650 PMC6559216

[B227] Shaheen M. R. Ayyub C. M. Amjad M. Waraich E. A. (2016). Morpho-physiological evaluation of tomato genotypes under high temperature stress conditions. *J. Sci. Food Agric.* 96 2698–2704. 10.1002/jsfa.7388 26304011

[B228] Shan-e-Ali Zaidi S. Vanderschuren H. Qaim M. Mahfouz M. M. Kohli A. Mansoor S. (2019). New plant breeding technologies for food security. *Science* 363 1390–1391. 10.1126/science.aav6316 30923209

[B229] Sharif B. Makowski D. Plauborg F. Olesen J. E. (2017). Comparison of regression techniques to predict response of oilseed rape yield to variation in climatic conditions in Denmark. *Eur. J. Agron.* 82 11–20. 10.1016/j.eja.2016.09.015

[B230] Sharma D. K. Torp A. M. Rosenqvist E. Ottosen C.-O. Andersen S. B. (2017). QTLs and potential candidate genes for heat stress tolerance identified from the mapping populations specifically segregating for Fv/Fm in wheat. *Front. Plant Sci.* 8:1668. 10.3389/fpls.2017.01668 29021798 PMC5623722

[B231] Shishkova S. Las Peñas M. L. Napsucialy-Mendivil S. Matvienko M. Kozik A. Montiel J. (2013). Determinate primary root growth as an adaptation to aridity in cactaceae: towards an understanding of the evolution and genetic control of the trait. *Ann. Bot.* 112 239–252. 10.1093/aob/mct100 23666887 PMC3698391

[B232] Singh L. P. Gill S. S. Tuteja N. (2011). Unraveling the role of fungal symbionts in plant abiotic stress tolerance. *Plant Signal. Behav.* 6 175–191. 10.4161/psb.6.2.14146 21512319 PMC3121976

[B233] Singh S. Kumar V. Kapoor D. Kumar S. Singh S. Dhanjal D. S. (2019). Revealing on hydrogen sulfide and nitric oxide signals co-ordination for plant growth under stress conditions. *Physiol. Plant* 168 301–317. 10.1111/ppl.13002 31264712

[B234] Song B. Song Y. Fu Y. Kizito E. B. Kamenya S. N. Kabod P. N. (2019). Draft genome sequence of Solanum aethiopicum provides insights into disease resistance, drought tolerance, and the evolution of the genome. *Gigascience* 8:115. 10.1093/gigascience/giz115 31574156 PMC6771550

[B235] Song J. Xing Y. Munir S. Yu C. Song L. Li H. (2016). An ATL78-Like RING-H2 finger protein confers abiotic stress tolerance through interacting with RAV2 and CSN5B in tomato. *Front. Plant Sci.* 07:1305. 10.3389/fpls.2016.01305 27621744 PMC5002894

[B236] Specht J. E. Hume D. J. Kumudini S. V. (1999). Soybean yield potential - A genetic and physiological perspective. *Crop Sci.* 39 1560–1570. 10.2135/cropsci1999.3961560x

[B237] Sturrock R. N. Frankel S. J. Brown A. V. Hennon P. E. Kliejunas J. T. Lewis K. J. (2011). Climate change and forest diseases. *Plant Pathol.* 60 133–149. 10.1111/j.1365-3059.2010.02406.x

[B238] Su Z. Tang Y. Ritchey L. E. Tack D. C. Zhu M. Bevilacqua P. C. (2018). Genome-wide RNA structurome reprogramming by acute heat shock globally regulates mRNA abundance. *Proc. Natl. Acad. Sci. U.S.A.* 115 12170–12175. 10.1073/pnas.1807988115 30413617 PMC6275526

[B239] Sun C. X. Gao X. X. Li M. Q. Fu J. Q. Zhang Y. L. (2016). Plastic responses in the metabolome and functional traits of maize plants to temperature variations. *Plant Biol.* 18 249–261. 10.1111/plb.12378 26280133

[B240] Suzuki N. Miller G. Morales J. Shulaev V. Torres M. A. Mittler R. (2011). Respiratory burst oxidases: the engines of ROS signaling. *Curr. Opin. Plant Biol.* 14 691–699. 10.1016/j.pbi.2011.07.014 21862390

[B241] Szabados L. Savouré A. (2010). Proline: a multifunctional amino acid. *Trends Plant Sci.* 15 89–97. 10.1016/j.tplants.2009.11.009 20036181

[B242] Tadele Z. Bartels D. (2019). Promoting orphan crops research and development. *Planta* 250 675–676. 10.1007/s00425-019-03235-x 31280328

[B243] Tai A. P. K. Martin M. V. Heald C. L. (2014). Threat to future global food security from climate change and ozone air pollution. *Nat. Clim. Chang.* 4 817–821. 10.1038/nclimate2317

[B244] Tai A. P. K. Val Martin M. (2017). Impacts of ozone air pollution and temperature extremes on crop yields: spatial variability, adaptation and implications for future food security. *Atmos. Environ.* 169 11–21. 10.1016/j.atmosenv.2017.09.002

[B245] Talanova V. V. Akimova T. V. Titov A. F. (2003). Effect of whole plant and local heating on the ABA content in cucumber seedling leaves and roots and on their heat tolerance. *Russ. J. Plant Physiol.* 50 90–94. 10.1023/A:1021996703940

[B246] Tanaka N. Kato M. Tomioka R. Kurata R. Fukao Y. Aoyama T. (2014). Characteristics of a root hair-less line of *Arabidopsis thaliana* under physiological stresses. *J. Exp. Bot.* 65 1497–1512. 10.1093/jxb/eru014 24501179 PMC3967087

[B247] Tang H. Takigawa M. Liu G. Zhu J. Kobayashi K. (2013). A projection of ozone-induced wheat production loss in China and India for the years 2000 and 2020 with exposure-based and flux-based approaches. *Glob. Chang. Biol.* 19 2739–2752. 10.1111/gcb.12252 23661338

[B248] Thiault L. Mora C. Cinner J. E. Cheung W. W. L. Graham N. A. J. Januchowski-hartley F. A. (2019). Escaping the perfect storm of simultaneous climate change impacts on agriculture and marine fisheries. *Science* 5:eaaw9976. 10.1126/sciadv.aaw9976 31807697 PMC6881155

[B249] Tigchelaar M. Battisti D. S. Naylor R. L. Ray D. K. (2018). Future warming increases probability of globally synchronized maize production shocks. *Proc. Natl. Acad. Sci. U.S.A.* 115 6644–6649. 10.1073/pnas.1718031115 29891651 PMC6042138

[B250] Tindall J. A. Mills H. A. Radcliffe D. E. (1990). The effect of root zone temperature on nutrient uptake of tomato. *J. Plant Nutr.* 13 939–956. 10.1080/01904169009364127

[B251] Torun H. (2019). Time-course analysis of salicylic acid effects on ROS regulation and antioxidant defense in roots of hulled and hulless barley under combined stress of drought, heat and salinity. *Physiol. Plant.* 165 169–182. 10.1111/ppl.12798 29984429

[B252] Trachsel S. Kaeppler S. M. Brown K. M. Lynch J. P. (2011). Shovelomics: high throughput phenotyping of maize (*Zea mays* L.) root architecture in the field. *Plant Soil* 341 75–87. 10.1007/s11104-010-0623-8

[B253] Trachsel S. Stamp P. Hund A. (2010). Effect of high temperatures, drought and aluminum toxicity on root growth of tropical maize (*Zea Mays* L.) seedlings. *Maydica* 55 249–260.

[B254] Tracy S. R. Nagel K. A. Postma J. A. Fassbender H. Wasson A. Watt M. (2020). Crop improvement from phenotyping roots: highlights reveal expanding opportunities. *Trends Plant Sci.* 25 105–118. 10.1016/j.tplants.2019.10.015 31806535

[B255] Uga Y. Sugimoto K. Ogawa S. Rane J. Ishitani M. Hara N. (2013). Control of root system architecture by DEEPER ROOTING 1 increases rice yield under drought conditions. *Nat. Genet.* 45 1097–1102. 10.1038/ng.2725 23913002

[B256] Ul Haq S. Khan A. Ali M. Khattak A. M. Gai W. X. Zhang H. X. (2019). Heat shock proteins: dynamic biomolecules to counter plant biotic and abiotic stresses. *Int. J. Mol. Sci.* 20:5321. 10.3390/ijms20215321 31731530 PMC6862505

[B257] Urban D. W. Roberts M. J. Schlenker W. Lobell D. B. (2015). The effects of extremely wet planting conditions on maize and soybean yields. *Clim. Change* 130 247–260. 10.1007/s10584-015-1362-x

[B258] Valdés-López O. Batek J. Gomez-Hernandez N. Nguyen C. T. Isidra-Arellano M. C. Zhang N. (2016). Soybean roots grown under heat stress show global changes in their transcriptional and proteomic profiles. *Front. Plant Sci.* 7:517. 10.3389/fpls.2016.00517 27200004 PMC4843095

[B259] van der Voort M. Kempenaar M. van Driel M. Raaijmakers J. M. Mendes R. (2016). Impact of soil heat on reassembly of bacterial communities in the rhizosphere microbiome and plant disease suppression. *Ecol. Lett.* 19 375–382. 10.1111/ele.12567 26833547

[B260] Van Dingenen R. Dentener F. J. Raes F. Krol M. C. Emberson L. Cofala J. (2009). The global impact of ozone on agricultural crop yields under current and future air quality legislation. *Atmos. Environ.* 43 604–618. 10.1016/j.atmosenv.2008.10.033

[B261] Van Inghelandt D. Frey F. P. Ries D. Stich B. (2019). QTL mapping and genome-wide prediction of heat tolerance in multiple connected populations of temperate maize. *Sci. Rep.* 9:14418. 10.1038/s41598-019-50853-2 31594984 PMC6783442

[B262] Vandermeiren K. Harmens H. Mills G. De Temmerman L. (2009). “Impacts of ground-level ozone on crop production in a changing climate,” *Climate Change and Crops. Environmental Science and Engineering*, ed. S. N. Singh (Berlin: Springer), 213–243. 10.1007/978-3-540-88246-6_10

[B263] Velásquez A. C. Castroverde C. D. M. He S. Y. (2018). Plant–pathogen warfare under changing climate conditions. *Curr. Biol.* 28 R619–R634. 10.1016/j.cub.2018.03.054 29787730 PMC5967643

[B264] Vishwakarma K. Upadhyay N. Kumar N. Yadav G. Singh J. Mishra R. K. (2017). Abscisic acid signaling and abiotic stress tolerance in plants: a review on current knowledge and future prospects. *Front. Plant Sci.* 8:161. 10.3389/fpls.2017.00161 28265276 PMC5316533

[B265] Vitousek S. Barnard P. L. Fletcher C. H. Frazer N. Erikson L. Storlazzi C. D. (2017). Doubling of coastal flooding frequency within decades due to sea-level rise. *Sci. Rep.* 7:1362. 10.1038/s41598-017-01362-7 28522843 PMC5437046

[B266] Vives-Peris V. Molina L. Segura A. Gómez-Cadenas A. Pérez-Clemente R. M. (2018). Root exudates from citrus plants subjected to abiotic stress conditions have a positive effect on rhizobacteria. *J. Plant Physiol.* 228 208–217. 10.1016/j.jplph.2018.06.003 30078434

[B267] Von Wettberg E. J. B. Chang P. L. Başdemir F. Carrasquila-Garcia N. Korbu L. B. Moenga S. M. (2018). Ecology and genomics of an important crop wild relative as a prelude to agricultural innovation. *Nat. Commun.* 9:649. 10.1038/s41467-018-02867-z 29440741 PMC5811434

[B268] Voss-Fels K. P. Snowdon R. J. Hickey L. T. (2018). Designer roots for future crops. *Trends Plant Sci.* 23 957–960. 10.1016/j.tplants.2018.08.004 30145109

[B269] Vu L. D. Gevaert K. De Smet I. (2019a). Feeling the heat: searching for plant thermosensors. *Trends Plant Sci.* 24 210–219. 10.1016/j.tplants.2018.11.004 30573309

[B270] Vu L. D. Xu X. Gevaert K. De Smet I. (2019b). Developmental plasticity at high temperature. *Plant Physiol.* 181 399–411. 10.1104/pp.19.00652 31363006 PMC6776856

[B271] Wahid A. Gelani S. Ashraf M. Foolad M. R. (2007). Heat tolerance in plants: an overview. *Environ. Exp. Bot.* 61 199–223. 10.1016/j.envexpbot.2007.05.011

[B272] Waines J. G. Ehdaie B. (2007). Domestication and crop physiology: roots of green-revolution wheat. *Ann. Bot.* 100 991–998. 10.1093/aob/mcm180 17940075 PMC2759207

[B273] Walter A. Liebisch F. Hund A. (2015). Plant phenotyping: from bean weighing to image analysis. *Plant Methods* 11:14. 10.1186/s13007-015-0056-8 25767559 PMC4357161

[B274] Wang H. Lemke R. Goddard T. Sprout C. (2007). Tillage and root heat stress in wheat in central Alberta. *Can. J. Soil Sci.* 87 3–10. 10.4141/S06-016

[B275] Wang H. Niu H. Liang M. Zhai Y. Huang W. Ding Q. (2019). A Wall-associated kinase gene CaWAKL20 from pepper negatively modulates plant thermotolerance by reducing the expression of ABA-responsive genes. *Front. Plant Sci.* 10:591. 10.3389/fpls.2019.00591 31156664 PMC6528620

[B276] Wang W. Qiu X. Yang Y. Kim H. S. Jia X. Yu H. (2019). Sweetpotato bZIP transcription factor IbABF4 confers tolerance to multiple abiotic stresses. *Front. Plant Sci.* 10:630. 10.3389/fpls.2019.00630 31156685 PMC6531819

[B277] Wang Y. Hu Z. Islam A. R. M. T. Chen S. Shang D. Xue Y. (2019). Effect of warming and elevated O3 concentration on CO2 emissions in a wheat-soybean rotation cropland rotation cropland. *Int. J. Environ. Res. Public Health* 16:1755. 10.3390/ijerph16101755 31108948 PMC6571970

[B278] Wang K. Zhang X. Goatley M. Ervin E. (2014). Heat Shock Proteins in relation to heat stress tolerance of creeping bentgrass at different N levels. *PLoS One* 9:e102914. 10.1371/journal.pone.0102914 25050702 PMC4106837

[B279] Wang R. Mei Y. Xu L. Zhu X. Wang Y. Guo J. (2018a). Differential proteomic analysis reveals sequential heat stress-responsive regulatory network in radish (*Raphanus sativus* L.) taproot. *Planta* 247 1109–1122. 10.1007/s00425-018-2846-5 29368016

[B280] Wang R. Mei Y. Xu L. Zhu X. Wang Y. Guo J. (2018b). Genome-wide characterization of differentially expressed genes provides insights into regulatory network of heat stress response in radish (*Raphanus sativus* L.). *Funct. Integr. Genomics* 18 225–239. 10.1007/s10142-017-0587-3 29332191

[B281] Wang W. Mauleon R. Hu Z. Chebotarov D. Tai S. Wu Z. (2018c). Genomic variation in 3,010 diverse accessions of Asian cultivated rice. *Nature* 557 43–49. 10.1038/s41586-018-0063-9 29695866 PMC6784863

[B282] Wang X. Zhuang L. Shi Y. Huang B. (2017). Up-regulation of HSFA2c and HSPs by ABA contributing to improved heat tolerance in tall fescue and *Arabidopsis*. *Int. J. Mol. Sci.* 18:1981. 10.3390/ijms18091981 28914758 PMC5618630

[B283] Waraich E. A. Ahmad R. Halim A. Aziz T. (2012). Alleviation of temperature stress by nutrient management in crop plants: a review. *J. Soil Sci. Plant Nutr.* 12 221–244. 10.4067/S0718-95162012000200003

[B284] Wasaya A. Zhang X. Fang Q. Yan Z. (2018). Root phenotyping for drought tolerance: a review. *Agronomy* 8:241 10.3390/agronomy8110241

[B285] Wei P. S. Chiu H. H. Hsieh Y. C. Yen D. L. Lee C. Tsai Y. C. (2019). Absorption coefficient of water vapor across atmospheric troposphere layer. *Heliyon* 5:e01145. 10.1016/j.heliyon.2019.e01145 30723826 PMC6351392

[B286] Wen J. Jiang F. Weng Y. Sun M. Shi X. Zhou Y. (2019). Identification of heat-tolerance QTLs and high-temperature stress-responsive genes through conventional QTL mapping, QTL-seq and RNA-seq in tomato. *BMC Plant Biol.* 19:398. 10.1186/s12870-019-2008-3 31510927 PMC6739936

[B287] White P. J. George T. S. Dupuy L. X. Karley A. J. Valentine T. A. Wiesel L. (2013). Root traits for infertile soils. *Front. Plant Sci.* 4:193. 10.3389/fpls.2013.00193 23781228 PMC3678079

[B288] Wigge P. A. (2013). Ambient temperature signalling in plants. *Curr. Opin. Plant Biol.* 16 661–666. 10.1016/j.pbi.2013.08.004 24021869

[B289] Winter D. Vinegar B. Nahal H. Ammar R. Wilson G. V. Provart N. J. (2007). An Electronic fluorescent pictograph browser for exploring and analyzing large-scale biological data sets. *PLoS One* 2:e718. 10.1371/journal.pone.0000718 17684564 PMC1934936

[B290] Wu X. Gong F. Cao D. Hu X. Wang W. (2016). Advances in crop proteomics: PTMs of proteins under abiotic stress. *Proteomics* 16 847–865. 10.1002/pmic.201500301 26616472

[B291] Wu Y.-S. Yang C.-Y. (2019). Ethylene-mediated signaling confers thermotolerance and regulates transcript levels of heat shock factors in rice seedlings under heat stress. *Bot. Stud.* 60:23. 10.1186/s40529-019-0272-z 31549254 PMC6757084

[B292] Xu C. Huang B. (2008). Root proteomic responses to heat stress in two Agrostis grass species contrasting in heat tolerance. *J. Exp. Bot.* 59 4183–4194. 10.1093/jxb/ern258 19008411 PMC2639019

[B293] Xu L. Zhang Q. Zhou A.-L. Huo R. (2013). Assessment of flood catastrophe risk for grain production at the provincial scale in China based on the BMM method. *J. Integr. Agric.* 12 2310–2320. 10.1016/S2095-3119(13)60587-0

[B294] Xue G.-P. Drenth J. McIntyre C. L. (2015). TaHsfA6f is a transcriptional activator that regulates a suite of heat stress protection genes in wheat (*Triticum aestivum* L.) including previously unknown Hsf targets. *J. Exp. Bot.* 66 1025–1039. 10.1093/jxb/eru462 25428996 PMC4321556

[B295] Yan Q. Duan Z. Mao J. Li X. Dong F. (2012). Effects of root-zone temperature and N, P, and K supplies on nutrient uptake of cucumber (*Cucumis sativus* L.) seedlings in hydroponics. *Soil Sci. Plant Nutr.* 58 707–717. 10.1080/00380768.2012.733925

[B296] Zaharieva M. Gaulin E. Havaux M. Acevedo E. Monneveux P. (2001). Drought and heat responses in the wild wheat relative *Aegilops geniculata* roth: potential interest for wheat improvement. *Crop Sci.* 41 1321–1329. 10.2135/cropsci2001.4141321x

[B297] Zandalinas S. I. Mittler R. Balfagón D. Arbona V. Gómez-Cadenas A. (2018). Plant adaptations to the combination of drought and high temperatures. *Physiol. Plant.* 162 2–12. 10.1111/ppl.12540 28042678

[B298] Zhai N. Jia H. Liu D. Liu S. Ma M. Guo X. (2017). *GhMAP3K65*, a cotton raf-like MAP3K gene, enhances susceptibility to Pathogen Infection and Heat Stress by Negatively Modulating Growth and Development in Transgenic *Nicotiana benthamiana*. *Int. J. Mol. Sci*. 18:2462. 10.3390/ijms18112462 29160794 PMC5713428

[B299] Zhang H. Yue M. Zheng X. Gautam M. He S. Li L. (2018). The role of promoter-associated histone acetylation of haem oxygenase-1 (HO-1) and giberellic acid-stimulated Like-1 (GSL-1) genes in heat-induced lateral root primordium inhibition in maize. *Front. Plant Sci.* 9:1520. 10.3389/fpls.2018.01520 30459784 PMC6232826

[B300] Zhou J. Wang J. Li X. Xia X.-J. Zhou Y.-H. Shi K. (2014). H2O2 mediates the crosstalk of brassinosteroid and abscisic acid in tomato responses to heat and oxidative stresses. *J. Exp. Bot.* 65 4371–4383. 10.1093/jxb/eru217 24899077 PMC4112640

[B301] Zhou R. Kong L. Wu Z. Rosenqvist E. Wang Y. Zhao L. (2019). Physiological response of tomatoes at drought, heat and their combination followed by recovery. *Physiol. Plant.* 165 144–154. 10.1111/ppl.12764 29774556

[B302] Zipper S. C. Qiu J. Kucharik C. J. (2016). Drought effects on US maize and soybean production: spatiotemporal patterns and historical changes. *Environ. Res. Lett.* 11:94021 10.1088/1748-9326/11/9/094021

[B303] Zörb C. Geilfus C. M. Dietz K. J. (2019). Salinity and crop yield. *Plant Biol.* 21 31–38. 10.1111/plb.12884 30059606

